# Genomic selection for growth and wood properties in multi-generation hybrid populations of *Populus deltoides*

**DOI:** 10.1093/hr/uhaf165

**Published:** 2025-06-25

**Authors:** Xinglu Zhou, Lei Zhang, Min Zhang, Hantian Wei, Yongxia Bai, Jinhong Tian, Jianjun Hu

**Affiliations:** State Key Laboratory of Tree Genetics and Breeding, Research Institute of Forestry, Chinese Academy of Forestry, No. 1 Dongxiaofu, Haidian District, Beijing 100091, China; Co-Innovation Center for Sustainable Forestry in Southern China, Nanjing Forestry University, No. 159 Longpan Road, Xuanwu District, Nanjing, Jiangsu 210037, China; State Key Laboratory of Tree Genetics and Breeding, Research Institute of Forestry, Chinese Academy of Forestry, No. 1 Dongxiaofu, Haidian District, Beijing 100091, China; Co-Innovation Center for Sustainable Forestry in Southern China, Nanjing Forestry University, No. 159 Longpan Road, Xuanwu District, Nanjing, Jiangsu 210037, China; State Key Laboratory of Tree Genetics and Breeding, Research Institute of Forestry, Chinese Academy of Forestry, No. 1 Dongxiaofu, Haidian District, Beijing 100091, China; State Key Laboratory of Tree Genetics and Breeding, Research Institute of Forestry, Chinese Academy of Forestry, No. 1 Dongxiaofu, Haidian District, Beijing 100091, China; State Key Laboratory of Tree Genetics and Breeding, Research Institute of Forestry, Chinese Academy of Forestry, No. 1 Dongxiaofu, Haidian District, Beijing 100091, China; State Key Laboratory of Tree Genetics and Breeding, Research Institute of Forestry, Chinese Academy of Forestry, No. 1 Dongxiaofu, Haidian District, Beijing 100091, China; State Key Laboratory of Tree Genetics and Breeding, Research Institute of Forestry, Chinese Academy of Forestry, No. 1 Dongxiaofu, Haidian District, Beijing 100091, China; Co-Innovation Center for Sustainable Forestry in Southern China, Nanjing Forestry University, No. 159 Longpan Road, Xuanwu District, Nanjing, Jiangsu 210037, China

## Abstract

The approximately 20-year breeding cycle has severely restricted the progress of genetic improvement in poplar. Genomic selection (GS) breeding has been demonstrated as an effective approach to accelerate this process. However, its application in forest tree species remains at an early stage. To advance the genetic improvement of target traits in *Populus deltoides*, the primary species of poplar plantations in China, we systematically implemented GS breeding using 765 hybrid progenies from 32 multi-generational full-sib families. Firstly, we assembled a high-quality genome of one core parent *P. deltoides* ‘Danhong’, with a genome size of 419.4 Mb and scaffold N50 of 22.0 Mb, which is also the first telomere-to-telomere (T2T) level genome of *P. deltoides*. Through comparative genomic analysis, we identified 1395 specific structural variants closely associated with growth and development. Subsequently, through genome-wide association studies (GWAS), we identified 135 quantitative trait nucleotides (QTNs) associated with growth and wood quality traits. By systematically evaluating reference genomes, statistical models, and various marker selection strategies, we developed optimal genomic prediction (GP) models for six traits, with the highest prediction accuracy (PA) reaching 0.730 for DBH. Compared with using all markers, the PA was improved by an average of 136.34%. Furthermore, by integrating GP, GWAS, and RNA-seq results, we identified core breeding parents and elite clones for *P. deltoides* genetic improvement and discovered important candidate genes. Our results provide a promising strategy for accelerating breeding cycles and genetic improvement, offering valuable breeding and genetic resources for forest tree improvement.

## Introduction

Poplar is a crucial timber species in China, and breeding high-yield, high-quality elite varieties has consistently been a critical task in poplar genetic improvement [1]. *Populus deltoides* exhibits rapid growth, high-quality wood, and strong resistance. Since its introduction in 1972, significant progress has been achieved in the genetic improvement and breeding of *P. deltoides* [2, 3], and the newly bred varieties have played a pivotal role in the development of industrial timber and ecological protection forests in China. Nevertheless, the imbalance between timber supply and demand in China persists as a pressing issue, with high annual timber imports [4]. In this context, there is an urgent imperative to accelerate forest tree genetic improvement to enhance genetic gains. Currently, the genetic gain of poplar primarily depends on traditional hybrid breeding, which is impeded by long breeding cycles and high costs, resulting in slow progress. Therefore, accelerating the breeding of superior poplar varieties to attain higher genetic gains holds significance.

The breeding 3.0 era of modern breeding methods and technologies assists traditional breeding has accelerated the genetic improvement process of forest trees [5]. Genomic selection (GS) has emerged as an effective breeding strategy since its introduction in 2001 [6], greatly reducing breeding cycles and enhances genetic gain compared to conventional methods. The accuracy of genomic prediction is typically evaluated using cross-validation, and populations with closer genetic relationships often exhibit better predictive ability [7, 8]. Moreover, prediction accuracy (PA) is influenced by multiple factors, such as statistical models, marker density, population structure, and population size. Improving PA has remained a focal point in GS breeding programs [9, 10].

**Figure 1:**
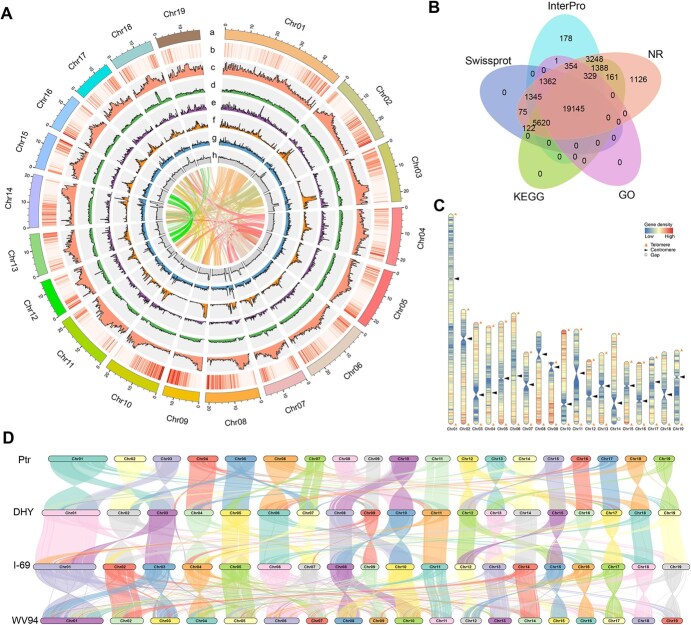
Genomic features, gene annotation, and synteny of *Populus deltoides* ‘Danhong’. (A) Gene distribution and repetitive sequence distribution across 19 chromosomes of DHY (window size: 200 kb). a represents the 19 chromosomes of DHY. b–h represent the distribution of genes, repetitive sequences, DNA repeats, Copia, Gypsy, TRF, and GC content, respectively. The inner circle illustrates the intra-genomic synteny of DHY genome. (B) Venn diagram of gene annotation in DHY. (C) Visualization of gene density, centromere, and telomere distribution in the DHY genome. (D) Collinearity analysis of DHY genome with *P. trichocarpa* v4.1 (Ptr), *P. deltoides* ‘I-69’ (I-69), and *P. deltoides* ‘WV94’ (WV94) genomes.

Establishing and optimizing the most appropriate model based on trait genetic structure is a crucial step in GS breeding programs [9]. As the core component of GS research, various classical statistical models have been developed, including GBLUP, RRBLUP, and Bayes series methods which are widely used [11]. Recently, machine learning and deep learning approaches have gradually been incorporated into GS breeding programs as novel strategies [12]. Currently, no model can be effectively applied to the majority of GS studies [6]. Apart from the model, marker density has been demonstrated to be another critical factor significantly influencing PA. For low-density markers, increasing marker density can frequently enhance PA [13]. However, for high-density markers obtained from whole-genome sequencing, the introduction of an excessive number of ineffective markers may give rise to the ‘large p, small n’ problem, resulting in model overfitting, and also wasting considerable computational resources [14]. Therefore, accurately selecting the optimal markers with high predictive power is a major challenge in conducting GS studies using high-density marker data. Genome-wide association studies (GWAS), as a method for identifying SNP markers associated with phenotypes, have been shown to enhance the efficiency of GS breeding [15]. Preliminary evidence has verified the feasibility of integrating GWAS with GS in species such as maize, soybean, and spruce [16, 17].

In fact, due to the unique biological characteristics of forest tree species and the limited basis of genetic research, GS studies in forest trees are still in the early stages. Currently, GS research in forest trees mainly focuses on high economically valuable species such as *Eucalyptus*, *Pinus*, *Picea*, and *Hevea* [18–20]. It is notable that, because of the perennial nature of forest species, the genetic gain of forest traits tends to be dynamic over time. Thus, evaluating the breeding potential of traits at maturity has more practical value [21]. However, this aspect is conspicuously lacking in GS research on forest tree. As a model species in forestry, systematic GS breeding research of poplars will provide essential reference value for the genetic improvement of forest trees [22].

Our research team has been actively engaged in multigenerational intraspecific hybridization breeding of *P. deltoides,* successfully cultivating several superior varieties such as *P. deltoides* ‘Chuangxin’, *P. deltoides* ‘Beiyang’, *P. deltoides* ‘Danhong’, and *P. deltoides* ‘Nanyang’ (Supplementary Fig. S1A). Among these, the female clone *P. deltoides* ‘Danhong’ (DHY) is notable for its fast growth, high yield, and strong resistance. It is widely cultivated in Southern China region, providing substantial economic and ecological benefits [3]. Based on these research background, and aiming at genetic improvement of growth and wood properties in *P. deltoides* plantations in China, we established a genomic selection reference and breeding population for *P. deltoides* using multi-generational full-sibling progenies, and systematically conducted genomic selection breeding research. At first, we assembled a high-quality telomere-to-telomere (T2T) level genome of the core parent DHY. Through GWAS analysis, we identified quantitative trait nucleotides (QTNs) and candidate genes related to growth and wood properties. Furthermore, we systematically evaluated the disparities in predictive ability among 14 GS models, investigated the influence of different marker screening strategies on model PA, and ultimately established the optimal GS prediction system for each trait. The primary objective is to disclose the genetic basis of important traits in *P. deltoides*, expedite the breeding process for superior varieties, and offer valuable genetic and breeding resources for the genetic improvement of forest trees.

## Results

### Sequencing, assembly, and evaluation of the DHY genome

Genome mapping rate assessments indicated that the existing poplar genomes are insufficient to meet the requirements for high-quality genetic analyses in multigeneration hybrid populations of *P. deltoides* (Supplementary Fig. S2). To ensure the high-quality implementation of the genomic selection (GS) program for *P. deltoides*, and considering the superior characteristics and central role of the cultivar *P. deltoides* ‘Danhong’ (DHY) in this study, we further assembled the DHY genome. Employing Illumina (58.05×), PacBio-Hifi (101.01×), and Hi-C (142.32×) sequencing methods, we generated a total of 122.99 Gb of raw sequence data (Supplementary Table S1). A genome survey indicated that the genome size was approximately 408.09 Mb, with a heterozygosity rate of 0.85% (Supplementary Fig. S3, Supplementary Table S2). Through integrating multiple assembly and correction approaches, we ultimately assembled the DHY genome (Fig. 1A, Supplementary Table S3), with a genome size of 419.4 Mb, a contig N50 and scaffold N50 lengths of 22.0 Mb each, and 95.5% of the sequences successfully anchored to 19 chromosomes (Table 1, Supplementary Table S4).

**Table 1:** Comparison of genomic characteristics of DHY, Ptr, I-69, and WV94

Assembly statistics	DHY	Ptr	I-69	WV94
Genome size	419.4 Mb	392.2 Mb	429.3 Mb	446.8 Mb
Number of chromosomes	19	19	19	19
Number of scaffolds	208	105	934	1375
Scaffold N50	22.0 Mb	13.2 Mb	21.5 Mb	21.7 Mb
Number of contigs	207	105	657	NA
Contig N50	22.0 Mb	13.2 Mb	2.6 Mb	0.6 Mb
GC percent	34.31	34.00	33.38	32.32
19 chromosomes (%)	95.5	95.1	97.4	90.2
Complete BUSCOs (%)	98.7	98.7	97.9	97.7
Repeat sequences of genome	45.22	46.33	47.30	NA
Gaps (%)	0.00	0.15	0.08	3.20

Note: Complete BUSCOs (%) were reassessed using the eudicot database (n = 2326).

Hi-C interaction heatmaps revealed no evident misassemblies across the assembled chromosomes (Supplementary Fig. S4). To further assess the quality of the assembled DHY genome, we estimated its continuity, completeness, sequence identity and accuracy. Except for chromosome 14 (with a 100-bp deletion), which was composed of two contigs, the remaining 18 chromosomes each consisted of only one contig. BUSCO analysis identified 98.7% of the conserved single-copy orthologs, reflecting a high level of gene completeness (Supplementary Fig. S5). The mapping rate was 99.31%, and the sequence accuracy reached 99.99% (Supplementary Tables S5 and S6). When compared with previously published *Populus* genomes, including Ptr [23], I-69 [24], and WV94 genome, the DHY genome exhibited superior quality in all assessment metrics (Table 1).

### Repetitive content and genome annotation

Based on multiple prediction approaches, approximately 189.12 Mb (45.22%) of repetitive sequences were identified in the entire genome (Supplementary Table S7). Long terminal repeats (LTRs) were the predominant transposable elements (TEs), including 13.65% LTR-Gypsy and 5.92% LTR-Copia. DNA transposable elements accounted for 19.92%, showing higher density at the ends of the chromosomes, which contrasts with the distribution of LTRs (Fig. 1A). By integrating *de novo* prediction, homology-based prediction and transcriptome-based prediction (Supplementary Fig. S6, Supplementary Tables S8 and S9), we annotated a total of 35 232 genes (Fig. 1A), of which approximately 97.79% were functionally annotations (Fig. 1B, Supplementary Table S10). We further annotated noncoding RNA genes (Supplementary Table S11), obtaining 6313 transfer RNA (tRNA) genes, 1820 ribosomal RNA (rRNA) genes, 731 microRNA (miRNA) genes and 598 small nuclear RNA (snRNA) genes.

**Figure 2:**
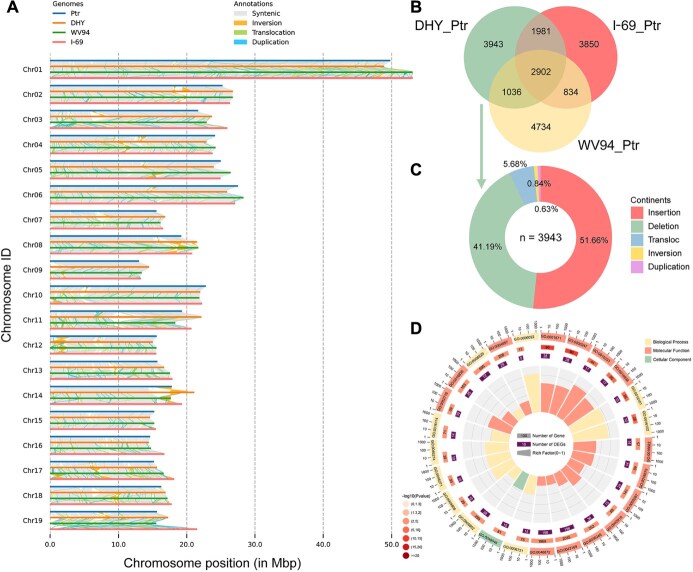
Global alignment analysis of DHY, Ptr, I-69, and WV94 using Ptr as the reference genome. (A) Large-scale structural variations among DHY, Ptr, I-69, and WV94 genome. (B) Venn diagram showing SVs. (C) Statistics of DHY-specific SVs. (D) GO enrichment analysis of genes associated with DHY-specific SVs.

### Identification of telomeres and centromeres

Using the plant seven-base telomeric repeat sequences (TTTAGGG/CCCTAAA) as the query, we predicted a total of 35 telomeres in the DHY genome (Fig. 1C), with the number of motif repeats ranging from 115 to 3211 (Supplementary Table S12). Among the 19 chromosomes, 16 chromosomes were assembled to the telomere-to-telomere (T2T) level, while the remaining three chromosomes only detected a single telomere. Except for chromosome 14, which has a single gap, all other chromosomes were assembled as gap-free sequences. Further, we also successfully predicted the centromeres of all chromosomes in the DHY genome (Fig. 1C). Therefore, the DHY genome can be regarded as a high-quality T2T-level assembly.

The total length of 19 centromeres was approximately 26 Mb, with an estimated length range from 0.12 to 3.84 Mb (Supplementary Table S13). The longest centromere was located on chromosome 11, while the shortest was on chromosome 6. The gene density of the centromeric regions was low, with a gene density of 7.12 genes/Mb, much lower than the genome average gene density of 84.09 genes/Mb. A total of 185 genes were annotated (Supplementary Table S14). GO and KEGG enrichment analyses indicated that these genes are primarily involved in processes closely related to plant growth and development (Supplementary Tables S15 and S16), suggesting that centromeric regions play an important role in plant growth and development.

### Whole-genome alignment and structural variations analysis

We performed collinearity analysis among the four genomes (DHY, Ptr, I-69, and WV94), revealing a high degree of synteny among the three *P. deltoides* genomes, while showing significant differences compared to the Ptr genome (Fig. 1D). Structural variations (SVs) play a crucial role in these differences. Using *P. trichocarpa* v4.1 as a reference, we identified 9862, 9567, and 9506 SVs (≥50 bp) in the DHY, I-69, and WV94 genomes, respectively. Among these SVs, the numbers of insertions and deletions were almost equal and significantly higher than the number of inversions or translocations (Supplementary Table S17).

Compared with the *P. trichocarpa* genome, large segmental SVs were observed in most chromosome sequences of *P. deltoides* genomes, particularly in Chr10, Chr12, and Chr14 (Fig. 2A). A total of 2902 SVs shared among the three *P. deltoides* genomes may represent species-specific differences between *P. deltoides* and *P. trichocarpa* (Fig. 2B). Among these, 1008 SVs were located within gene regions, affecting the structure of 1658 *P. trichocarpa* genes (Supplementary Table S18). These genes are primarily involved in glutathione metabolic process, terpene metabolic process, methyl jasmonate esterase activity, ion transmembrane transport, photosynthesis, and purine metabolism (Supplementary Fig. S7).

DHY genome contains 3943 unique SVs, including 2037 INS, 1624 DEL, 25 DUP, 33 INV, and 224 TRAN (Fig. 2C, Supplementary Table S19). Among these, 1395 INS and DEL type SVs were located within gene regions, affecting the structure of 2875 DHY genes (Supplementary Table S20). Some of these genes, such as *IAA26* (Pde01g02130), *ARF22* (Pde15g00146), *GRF10* (Pde03g00929), *ARR17* (Pde19g00001), *ABA2* (Pde05g01122), and *CDKF-4* (Pde01g03485), were closely related to defense response, response to stress, response to stimuli, abscisic acid binding, growth, and rhythmic processes (Fig. 2D). These genes may play an important role in the rapid growth and high insect resistance of *P. deltoides* ‘Danhong’.

### Population genotyping and population structure

Whole-genome resequencing of 765 trees from the reference and breeding populations yielded a total of 4.38 Tb of raw data (Supplementary Table S21), with an average sequencing depth of 11.74×. Mapping rates of these resequencing data differed significantly among the DHY, Ptr, I-69, and WV94 genome assemblies. Specifically, the average mapping rate of the resequencing data in the DHY genome was 97.69%, which was significantly higher than that obtained with the Ptr (89.04%), I-69 (91.21%), and WV94 (94.80%) genomes (Supplementary Fig. S2). This suggests that the DHY genome is more appropriate as a reference genome for subsequent studies. Subsequently, with the DHY genome as a reference, approximately 1.14 million high-quality SNPs were identified in 765 trees after strict quality control and filtering. These SNPs were uniformly distributed across the 19 chromosomes, with an average density of approximately 2845 SNPs/Mb (Supplementary Fig. S8).

**Figure 3:**
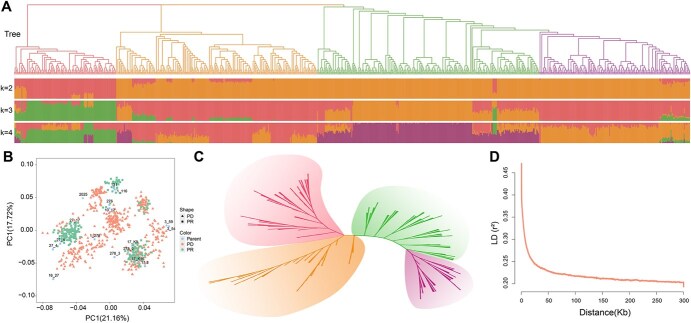
Population genetic structure and principal component analysis. (A) Phylogenetic tree and population structure of 765 individuals, divided into four groups, with different colors representing different groups. (B) PCA plot of 765 individuals. (C) Unrooted phylogenetic tree of the reference population, showing a similar genetic structure to the entire population, also divided into four groups. (D) LD decay plot for the reference population, with the LD decay distance around 16 kb, used as the interval for identifying candidate genes in GWAS analyses.

Population structure analysis and the neighbor-joining phylogenetic tree divided the 765 trees into four clusters (Fig. 3A). Cluster 1 presented the most complex genetic background, showing an intermediate type among various cross combinations. Cluster 2 was genetically inclined towards hybrid parent *P. deltoides* ‘Danhong’ and *P. deltoides* ‘2025’, Cluster 3 towards hybrid parent *deltoides* ‘Danhong’ and *deltoides* ‘Beiyang’, and Cluster 4 towards hybrid parent *P. deltoides* ‘Zhongcheng2’ and *P. deltoides* ‘Chuangxin’. The PCA results were in accordance with the findings of the population structure and phylogenetic tree (Fig. 3B). The entire population did not show significant stratification, and the reference population exhibited a similar genetic structure to that of the breeding population (Fig. 3B and C), which facilitated the smooth conduct of subsequent genomic selection studies.

### Trait variation, heritability, and correlation

Based on the genetic improvement goals, we collected data regarding tree height (H), diameter at breast height (DBH), basic density (BD), microfibril angle (MA), fiber length (FL), and fiber width (FW) from the reference population. All six traits demonstrated approximately normal distributions (Supplementary Table S22, Supplementary Fig. S9), with coefficients of variation (CV) ranging from 6.42% to 18.94%. The heritability (H^2^) of six traits ranged from 0.13 to 0.87, with DBH and BD presented relatively high heritability, both exceeding 0.8, whereas MA and FL showed lower heritability, at 0.13 and 0.14, respectively (Table 2). Pearson correlation analysis disclosed a low correlation between growth traits and wood properties (Supplementary Fig. S9), indicating that it might be challenging to select for both growth traits and wood properties simultaneously under conventional breeding conditions.

**Table 2:** Summary statistics and heritability (H^2^) of phenotypic data

Trait	Mean	Standard deviation	Maximum	Minimum	Coefficient of variation	Heritability
DBH (cm)	22.24	4.21	33.56	12.04	18.94%	0.87
H (m)	14.37	1.38	18.08	10.06	9.60%	0.42
BD (g/cm^3^)	0.41	0.03	0.51	0.32	6.97%	0.80
MA (°)	14.21	0.91	17.81	11.96	6.42%	0.13
FL (μm)	1147.45	78.07	1308.26	896.10	6.80%	0.14
FW (μm)	24.60	2.28	31.16	18.56	9.28%	0.24

### GWAS and candidate gene identification

**Figure 4:**
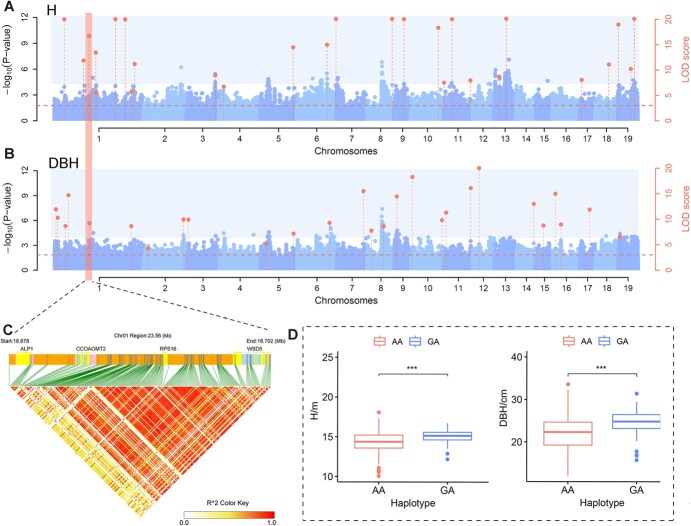
Genome-wide association studies for growth traits and identification of significant SNPs. (A) and (B) Manhattan plots for genome-wide association studies of tree height (H) and diameter at breast height (DBH), respectively. The left Y-axis shows the -log10 P values of all SNP obtained from the first step of single-marker scanning; the right Y-axis shows the LOD values of all significant and suggestive loci from the second step. The dashed line indicates the threshold for the second round of detection using the IIIVmrMLM model (LOD = 3). Highlighted points within the shaded regions represent significant QTNs, while those outside the shaded areas indicate suggested QTNs. (C) Linkage disequilibrium (LD) within the co-localized QTN regions for H and DBH. (D) Haplotype analysis bar plot for co-localized QTNs of H and DBH, with ^***^ indicating *P* < 0.001.

To identify QTNs and candidate genes associated with traits, based on phenotypic data and the genotypic information of the reference population, we conducted GWAS using the IIIVmrMLM software. A total of 135 QTNs, comprising 116 significant QTNs and 19 suggested QTNs, were identified for the six traits (Fig. 4A and B; Supplementary Fig. S10; Supplementary Table S23). The logarithm of odds (LOD) values of significant QTNs ranged from 3.879 to 47.029, and the phenotypic variance explained (PVE) ranged from 0.687% to 6.999% (Supplementary Table S23). Using LD decay distance (16 kb) as the candidate gene selection interval (Fig. 3D), a total of 195 functionally annotated genes were identified in the DHY genome (Supplementary Table S24). By integrating published GWAS studies on growth and wood properties in poplar, a total of 17 prior candidate genes were identified. Among these, five genes were identified in at least two studies, with *RGA2* and *RNP1* identified in four studies (Supplementary Table S25).

Within the LD decay region of the QTN Chr01_18689600, which is co-localized with both H and DBH, four genes were identified (Fig. 4C). Among them, two genes are notable: *RPS16* is involved in protein synthesis, maintaining essential cellular activities and growth; *CCOAOMT2* participates in lignin biosynthesis, though its potential indirect role in poplar growth remains to be verified. T-tests analysis indicated that QTN Chr01_18689600 showed significant differences among the different haplotypes (Fig. 4C), with heterozygous haplotypes presenting notable advantages (Fig. 4D). Based on gene annotation and enrichment analysis, 18 candidate genes potentially related to target traits were initially identified (Supplementary Table S24). These include *INVE*, *ROPGEF8*, *TAP2*, *PCMP*, and *ADF6*, which are associated with plant cell wall synthesis; *MYB105*, *MYB113*, and *CCOAOMT2*, which are involved in lignin biosynthesis; *LHCB7*, *FDC1*, and *TOC159*, related to photosynthesis; *ERF053*, *WRKY27*, *HSP70*, *PIL1*, and *TPS11*, associated with stress response; and *KCS7* and *GAF1*, related to plant hormones.

### Evaluation of genomic selection statistical models

**Figure 5:**
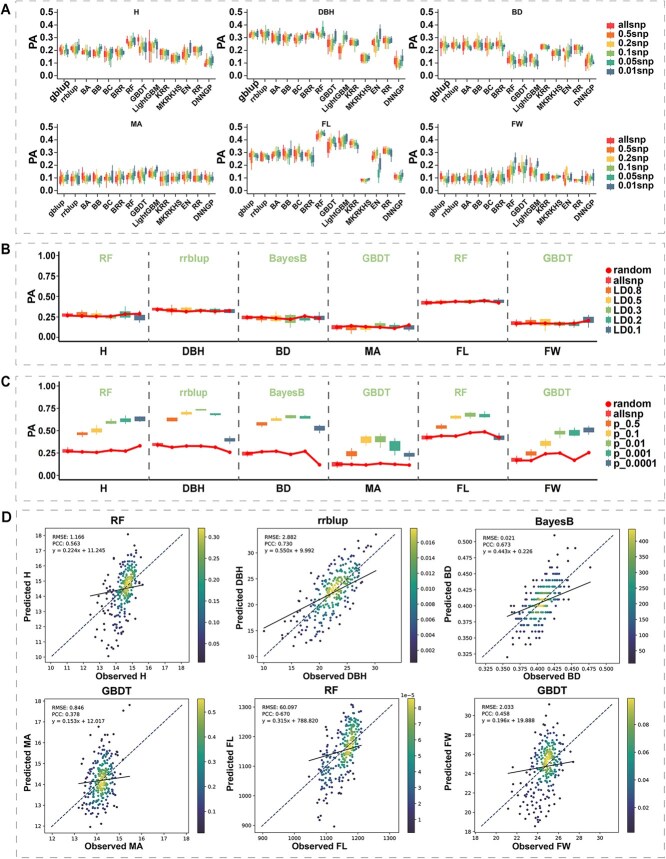
Differences in predictive ability of different GS models and the impact of different marker selection strategies on PA. (A) Predictive ability of 14 GS models for six traits of *P. deltoides* at different marker densities. (B) Effect of LD levels between markers on PA. (C) Effect of marker-phenotype association levels on PA, with dots representing PA under randomly selected markers. (D) Density plots showing prediction accuracy of the optimal model for each trait.

To ensure efficient prediction in genomic selection (GS), we first conducted a systematic evaluation of the predictive abilities of 14 different GS statistical models for the genetic improvement traits and identified the optimal statistical model for each trait. The results demonstrated significant differences in prediction accuracy (PA) among the different models at the same marker density. However, no significant differences in PA were observed when the same model was tested across various marker densities (Fig. 5A, Supplementary Table S26). The traditional BLUP-based methods and Bayesian methods displayed similar prediction abilities, whereas machine learning models exhibited better PA for complex traits with low heritability (Fig. 5A). Based on the combined predictive performance across six marker densities, we identified the optimal model for each trait. The optimal statistical model for H was RF (0.266), for DBH was rrBLUP (0.323), for BD was BD (0.244), for MA was GBDT (0.126), for FL was RF (0.438), and for FW was GBDT (0.178). Overall, the PA for each trait remained relatively low across the six random marker selection strategies, with DBH showing the highest accuracy and MA the lowest.

Subsequently, based on the optimal model for each trait, we compared the impacts of commonly used cross-validation methods on PA. The results indicated that different cross-validation methods had a minimal effect on model prediction accuracy, though there were differences in model prediction runtime. Leave-one-out cross-validation (LOOCV) required the longest prediction time, followed by 10-fold cross-validation. In contrast, five-fold cross-validation had the shortest computation time and yielded the most stable PA, exhibiting less variability across trials (Supplementary Fig. S11).

### Impact of different marker selection strategies on the PA

To determine the optimal subset of predictive markers for each trait, we investigated the effects of different LD levels and marker-trait associations on model prediction accuracy (PA), using randomly selected markers as a control. The results demonstrated no significant difference in the model’s PA at different LD levels compared to models using an equivalent number of random markers, with PA being most stable when the LD level matched the population’s linkage structure (Fig. 5B, Supplementary Table S27). For marker-trait associations, significant differences in PA across traits were observed at different *P*-value thresholds, and significant differences were also found compared to models using an equivalent number of random markers. For the traits of DBH, BD, MA, and FL, PA initially increased and then decreased with higher marker-trait correlation, while H and FW showed an increasing and stabilizing trend (Fig. 5C, Supplementary Table S28).

By integrating the PA from different marker selection strategies, we discovered that each trait demonstrated better prediction performance when the marker-trait correlation reached the 0.01 level. The final PA for the six traits (H, DBH, BD, MA, FL, and FW) were 0.563, 0.730, 0.673, 0.378, 0.670, and 0.458, respectively (Fig. 5D). The Pearson correlation coefficient between prediction accuracy and trait heritability was 0.669. Compared with the PA using all markers, the PAs for each trait increased by 89.56%, 115.34%, 178.10%, 202.40%, 58.39%, and 174.25% (Supplementary Table S29), respectively, highlighting the significance of marker selection in enhancing PA, with an average improvement of 136.34%.

### Multi-trait genomic selection breeding

**Figure 6:**
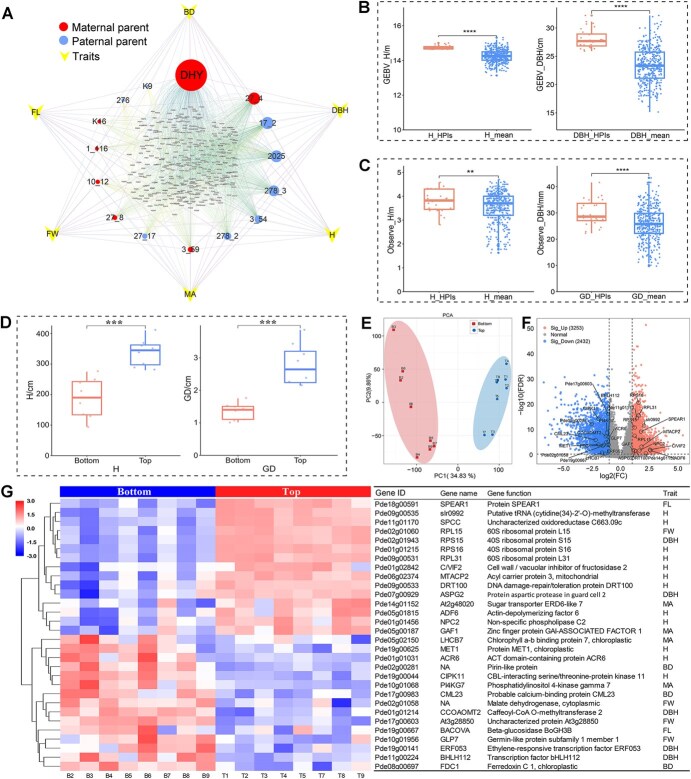
Selection of core parents and transcriptomic analysis of extreme individuals. (A) Kinship networks of superior hybrid progenies for different traits. For each trait, the top 20% of hybrid progenies exhibiting superior performance were selected. Kinship networks were then constructed based on the parental information of these superior hybrids, and node weights were calculated. Node size indicates the number of superior individuals associated with each parent. (B) and (C) Genetic gains of high-performing individuals (HPIs) relative to the population mean, where ^**^ indicates significance at the 0.05 level, and ^****^ indicates significance at the 0.0001 level. (D) Statistical results of seedling growth traits for top and bottom extreme individuals, where ^***^ indicates significance at the 0.001 level. (E) PCA clustering of transcriptome samples, showing two distinct groups. (F) Volcano plot of differentially expressed genes (DEGs) between bottom and top individuals, with labeled genes representing overlaps with GWAS candidate genes. (G) Heatmap of expression levels for overlapping genes across different.

Employing the optimal statistical model and marker subset for each trait, we estimated the genomic estimated breeding values (GEBV) for the breeding population. The results indicated that the GEBVs for all traits followed a normal distribution (Supplementary Fig. S12, Supplementary Table S30). We adopted a 20% selection rate to identify superior clones for each trait. By integrating the kinship network information of the superior clones, we further identified the top-performing maternal clones for the genetic improvement of *P. deltoides*, including *P. deltoides* ‘Danhong’ (131), *P. deltoides* ‘27–4’ (27–4), and *P. deltoides* ‘Zhongcheng 3’ (3–59), as well as the superior paternal clones *P. deltoides* ‘Chuangxin’ (17–2), *P. deltoides* ‘2025’ (2025), and *P. deltoides* ‘Zhongcheng 4’ (3–54) (Fig. 6A).

By combining the GP results for H and DBH, we screened 28 superior clones with significantly higher GEBVs and field observations than the population average. The genetic gains in GEBV for H and DBH were 9.76% and 22.02% above the mean, respectively (Fig. 6B), while actual seedling growth gains were 7.29% and 16.53%, respectively, providing preliminary confirmation of the reliability of GP predictions (Fig. 6C). These superior clones can be utilized not only for biomass energy development but also as key breeding materials in the genetic improvement of *P. deltoides*. For example, when the breeding objective focuses on producing construction materials or furniture, wood strength can be enhanced by increasing basic wood density. Based on GEBV predictions for wood density, we selected three superior clones (PD5, PD464, and PD474) for this purpose. Conversely, when the breeding objective is pulp and paper production, pulp yield can be improved by reducing basic density and increasing fiber length. According to the predicted basic density and fiber length, six clones (PD87, PD311, PD465, PD468, PD474, and PD488) were selected for pulp production.

### Transcriptome analysis of growth extreme individuals based on GP

Based on the genomic composite breeding values (GCBV) for growth traits and seedling growth data, we selected eight high-performing clones (Top) and eight low-performing clones (Bottom) for transcriptome analysis. The genetic backgrounds of these clones are detailed in Supplementary Table S31. Significant differences in seedling growth traits were observed between the Top and Bottom groups, with the average tree height in the Top group being approximately 1.79 times that of the Bottom group, and the ground diameter was about 2.03 times greater (Fig. 6D). Hierarchical clustering heatmaps and principal component analysis (PCA) of expression data further confirmed that the 16 samples could be broadly classified into two distinct groups (Supplementary Fig. S13, Fig. 6E).

Differential expression analysis disclosed a total of 5685 differentially expressed genes (DEGs) between the two groups (Fig. 6F), of which 5402 were mapped to annotated genes in the reference genome, while the remaining 283 were novel genes identified through transcript annotation. These DEGs were primarily related to growth, development, carbon utilization, and significantly enriched in pathways concerning amino acid metabolism, secondary metabolite biosynthesis, and plant circadian rhythm (Supplementary Fig. S14). Notably, among the genes associated with DHY genome-specific structural variations, 383 genes were differentially expressed (Supplementary Table S20). In addition, 30 of the GWAS candidate genes were differentially expressed (Supplementary Table S32), with 15 genes upregulated and 15 genes downregulated (Fig. 6G). In our previous analysis, the co-localized candidate genes *CCOAOMT2* and *RPS16* for DBH and H were both differentially expressed between the two groups. Specifically, *RPS16* was upregulated, with correlation coefficients of 0.87 and 0.91 for gene expression levels with tree height and DBH, respectively. *CCOAOMT2* was downregulated, with correlation coefficients of −0.71 and −0.83 for tree height and DBH, respectively (Fig. 6G). Based on the combined results from GP prediction, GWAS, and RNA-seq analysis, we contend that *RPS16*, *CCOAOMT2*, *ERF053*, and *BHLH112* are worthy of further exploration and will be investigated in subsequent studies.

## Discussion

High-quality genomes have long been a central objective in genomics research. With the advancement of third-generation sequencing technologies, an increasing number of species are achieving telomere-to-telomere (T2T) genomic assemblies. However, T2T-level genomic research in poplars remains relatively scarce [25, 26], and there is currently no T2T-level genome available for *P. deltoides.* T2T-level genome assembly strategies mainly rely on the combination of HiFi, ONT, and Hi-C technologies [27]. Comprehensive assembly approaches involving high-depth HiFi and Hi-C have also enabled the construction of T2T-level genomes in multiple species [28–30]. In this study, we integrated high-depth HiFi and Hi-C data to achieve T2T-level genome assembly for *P. deltoides* ‘Danhong’ (DHY). Compared with previously reported poplar genomes such as I-69 and WV94, the DHY genome exhibited superior metrics across various aspects, and filled gaps in centromeric information in current *P. deltoides* genomes. It is noteworthy that the anchoring rates of the 19 chromosomes in the DHY, Ptr, and WV94 genomes are slightly lower than that of the I-69 genome, primarily because potentially highly heterozygous sequences in the I-69 genome were filtered out before being anchored to the I-69 genome (Bai et al., 2021). While such filtering may improve assembly clarity, it can result in the loss of valuable genetic variation. Given the complexity of poplar genomes and the significance of structural variations (SVs) in genomic studies, the development of a poplar pan-genome will be indispensable. In previous research, Wang et al. [31] reported a pan-genome study of the *Populus* genus, advancing the understanding of genetic variation in poplars. For multi-generational genomic selection breeding, constructing a pan-genome involving multiple parents holds even greater breeding value. Undoubtedly, the T2T-level DHY genome will serve as an important reference for *P. deltoides* pan-genome research, and will accelerate the breeding process of poplar genomic selection.

Genomic selection (GS) breeding, as an emerging breeding strategy, has been extensively utilized in animal and crop breeding. It has successfully reduced the breeding cycle of dairy cattle from 7 years to 1 year, resulting in a 35% increase in genetic gains [32, 33], and increased rice yield by 16% [34]. However, GS research in forestry has advanced relatively slowly [18, 33, 35]. As a model species for forest trees, conducting systematic GS research on poplar holds significant reference value for promoting GS in forestry [36]. The primary direction of forest tree genetic improvement is to breed new varieties that combine both growth and wood quality traits. However, these traits often interact in practical breeding, resulting in fast-growing varieties that may be accompanied by decreased wood density and excessive fiber length [18]. This interaction is a key reason why conventional breeding methods for poplars struggle to simultaneously improve both growth and wood quality traits. How to harness the advantages of GS to achieve early selection for multiple trait pyramiding will be a key focus of future research in forest tree GS. Choosing an appropriate statistical model is crucial, as different models incorporate varying assumptions about marker effects [37], leading to differences in PA. In many previous GS studies, models were selected empirically without rigorous evaluation. Here, we systematically compared the prediction accuracy (PA) of 14 commonly used GS models. For the same trait, PA varied significantly across models, and the variation was even more pronounced across traits. Machine learning models, particularly RF and GBDT, demonstrated advantages in predicting traits with low heritability, whereas BLUP-like and Bayes-like methods exhibited relatively stable performance across different traits.

The successful application of GS breeding depends largely on the accuracy of genomic prediction (GP) [38]. Previous studies have indicated that model PA approached zero as the genetic relationship between populations weakened [39, 40]. In this study, we constructed reference and breeding populations based on multigenerational hybridization, and the similar genetic structure and relationships between these populations guaranteed the accuracy of the GP. In addition, optimizing marker density is also beneficial for improving the accuracy of GP. The optimal density and distribution of SNP markers generally depend on the number of QTNs controlling the target trait and the LD level [6]. Weber et al. [41] reported that using haplotype blocks rather than single SNP markers can effectively improve GP accuracy. Under conditions of high marker density, PA is less affected by marker density. However, when the number of markers decreases sharply, the PA begins to decline. In our study, we found no significant differences in PA among different models across six LD levels. We speculate that this may be because, even at the lowest LD threshold, the number of markers remained relatively high, thus maintaining stable prediction accuracy.

Although the primary focus of GP is on prediction accuracy, computational time is also an important factor. In our study, running 10 iterations of five-fold cross-validation using all markers (approximately 1.14 million) required between 88.25 and 161.77 minutes for BLUP and Bayes-type models, with an average time of about 110.57 minutes. In contrast, after LD-based optimization (74 770 SNPs), the average computation time was only about 2.45 minutes, representing a 45.13-fold reduction in computational time, with no significant difference in prediction accuracy compared to using all markers. Combined with the view of Xu et al. [8], we suggest that for high-density marker GS studies, filtering markers based on population LD levels can significantly reduce computational costs while maintaining stable PA. It is worth noting that this conclusion is based on a hybrid population, and its applicability to natural populations still needs further investigation. Afterward, we compared their runtime using the LD-optimized markers (74 770 SNPs). The RR model had the shortest runtime, while models such as GBLUP, EN, RR, DNNGP, and KRR had similar computation times. In contrast, BA, BB, MKRKHS, and RF models required longer runtimes (Supplementary Fig. S15). Of course, these results are only suitable for comparison purposes, as they are highly dependent on the characteristics of the bioinformatics server. In our study, computations were performed on a server equipped with AMD EPYC 7763 * 2 CPUs (64 cores, 128 threads) and 1024 GB memory.

Previous studies have demonstrated that fully accounting for correlation between genetic markers and traits can enhance the prediction accuracy of GS models [42]. Consistent with most studies [43–45], the GWAS-assisted GS approach in this study resulted in a substantial improvement in PA. Specifically, we found that using the top 1% of markers significantly associated with the trait yielded the best prediction results for nearly all traits, with an average prediction efficiency increase of 136.34%. Compared to previous GS studies in forest trees, the PA for each trait was at a relatively high level, with a similar correlation between accuracy and trait heritability (*r* = 0.669) [18, 22, 35, 46]. Based on this, using GWAS results as the basis for selection will help improve the PA of the model, further enable high-precision early selection of superior clones, and ultimately provide a guarantee for multi-trait pyramiding breeding.

Beyond PA improvement, GWAS also provides candidate genes for molecular breeding. Currently, the majority of GWAS study populations consist of natural populations, primarily because these populations have undergone extensive historical variation and genetic diversity [47]. However, natural forest populations are often sparsely distributed and have experienced limited gene flow over time [48], which can increase the probability of false positives in QTL linkage associations. For biparental hybrid populations, limited genetic diversity is generally regarded as constraining the feasibility of GWAS studies. In contrast, multiparent hybrid populations exhibit rich phenotypic variation due to the genetic differences among parents, allowing for the exploration of genetic recombination effects on trait variation and increasing the frequency of rare mutation sites [21, 49, 50]. Furthermore, hybrid populations have clear pedigree information, which can effectively reduce marker error rates. The feasibility of GWAS studies in hybrid populations has been demonstrated in multiple tree species, including *Macadamia integrifolia* [50], *Elaeis oleifera* × *E. guineensis* [49], and *Liriodendron chinense* [21, 51]. In this study, we identified multiple candidate genes associated with growth and wood properties through GWAS in a multi-generational hybrid population. By integrating published GWAS studies on growth and wood properties in poplar, a total of 19 prior candidate genes were identified. Among these, five genes were detected in at least two studies, with *RGA2* and *RNP1* identified in four studies. Those results further highlighting the necessity and feasibility of conducting GWAS in hybrid populations.

In summary, we report a high-quality T2T genome assembly of *P. deltoides* ‘Danhong’, filling the research gap in the centromeric regions of poplar genomes. Through large-scale whole-genome resequencing, we identified key candidate genes associated with growth and wood properties, and established an optimal genomic selection method for these traits. Furthermore, this work demonstrated the feasibility of integrating GS, GWAS, and transcriptomics in poplar genetic improvement, offering a promising approach to shorten breeding cycles and achieve targeted genetic enhancement.

## Materials and methods

### Genomic selection population and phenotypic data collection of *Populus deltoides*

The genomic selection (GS) population comprises a reference population and a breeding population, comprising a total of 765 hybrid progenies from 32 multigenerational full-sib families. The reference population consists of 260 trees, selected from five full-sibling families created through artificial hybridization, which were planted in 2014 at the Daxing Forest Farm, Beijing. In these families, the female parents were *P. deltoides* ‘Danhong’, while the male parents were selected superior *P. deltoides* varieties. Several rows of shelterbelts were planted around the trial plantation to minimize the influence of edge effects and other microenvironmental factors. The breeding population consists of 505 trees, selected from over 2500 hybrid progenies obtained through artificial hybridization during 2021 and 2022. These progenies were derived from 27 full-sibling families established based on the genetic background of the reference population’s parents. Genetic background of the parents and the number of progenies per family are provided in Supplementary Fig. S1A and B. The reference population and breeding population were asexually propagated in three locations: Haidian District, Beijing; Heze City, Shandong Province; and Jiaozuo City, Henan Province.

From 2021 to 2024, tree height and diameter at breast height (DBH) were collected from the reference population every March. Seedling growth data were measured in December 2023. In December 2021, increment cores were extracted from the DBH of each tree using an increment borer, from south to north. To guarantee the integrity of the annual rings, the outermost ring was removed, and the second-to-last annual ring was selected for measurements of basic density, microfibril angle, fiber length, and fiber width. The measurement methods adhered to national standard procedures.

### Genome sequencing, genome assembly, and genome quality assessment

Fresh leaf tissue of *P. deltoides* ‘Danhong’ was collected, and high-quality genomic DNA was extracted using a modified CTAB method. High-depth sequencing was carried out using three different sequencing technologies: Illumina (Illumina, San Diego, CA, USA), PacBio-Hifi (Pacific Biosciences, San Diego, CA, USA), and Hi-C. Sequencing libraries were constructed following the sample preparation guide, and sequencing data were utilized for genome assembly after quality control based on the method described by Li et al. [52].

The genome size and heterozygosity of DHY were evaluated using K-mer analysis (K = 17) based on the Illumina data. Subsequently, genomic contig assembly was conducted using Hifiasm [53] based on PacBio-Hifi sequencing data. Next, mitochondrial and chloroplast sequences of genomic contig sequences were filtered out using Minimap2 [54], and the remaining genomic contig sequences were anchored to chromosomes using ALLHiC [55]. Finally, corrections were manually made to the genome based on chromosomal interaction intensity using Juicebox [56].

Genome continuity was assessed by determining the number of contigs, N50 length, and the number of gaps. Genome completeness was evaluated using 2326 core eukaryotic genes from the Benchmarking Universal Single-Copy Orthologs (BUSCO) database (http://busco.ezlab.org). Sequence consistency and accuracy were examined using BWA [57] and Merqury [58] .

### Telomere and centromere identification

Telomeres were identified in the terminal 100 kb of each chromosome in the DHY genome by using plant-specific telomeric sequences (TTTAGGG/CCCTAAA). Telomere detection was conducted using quarTeT [59] with default parameters. Centromeres were predicted using Tandem Repeat Finder (TRF) [60], taking into account the position of repeat sequences, with the TRF parameters set to 2 7 7 80 10 50 200. Further, gene annotation within centromeric regions was carried out using bcftools [61], and Gene Ontology (GO) and Kyoto Encyclopedia of Genes and Genomes (KEGG) enrichment analyses were performed using the R package ‘clusterProfiler’ [62].

### Genome annotation, and functional prediction

Genome annotation mainly consisted of repetitive sequence annotation, gene annotation, and non-coding RNA annotation. Repetitive sequence annotation employed two approaches: homology-based sequence alignment and de novo prediction. Sequence alignment relied on the RepBase database (http://www.girinst.org/repbase) using Repeatmasker [63] for directly predicted. *De novo* prediction utilized RepeatModeler [64] to initially build a de novo repetitive sequence library, followed by annotation with RepeatMasker. For gene annotation, gene structure prediction was initially performed using de novo prediction, homology-based prediction, and RNA-seq-based prediction. *De novo* prediction was carried out using Augustus [65] and SNAP [66]. Homology-based prediction used gene models from *Arabidopsis thaliana*, *P. trichocarpa*, and *P. deltoides* and conducted using BLAST [67] and GeneWise [68]. RNA-seq prediction relied on transcriptome data from *P. deltoides* ‘Danhong’, using GeneMarkS-T [69] for predicting gene structures from the assembled transcripts. The gene structure predictions from these different methods were integrated and refined using EVidenceModeler (EVM) [70] and PASA [71]. Finally, the predicted protein sequences were aligned against the SwissProt, InterPro, Nr, KEGG, and GO databases to obtain functional annotation for all predicted genes. Noncoding RNA annotation was conducted using Infernal [72] to predict miRNA and snRNA, tRNA using tRNAscan-SE [73], and rRNA using BLAST.

**Table 3:** The 14 genomic selection statistical models used in this study

Model category	Model name	Abbreviation	Evaluation method	Running environment
BLUP Class	Genomic best linear unbiased prediction	GBLUP	5-Fold CV	R package ‘sommer’
	Ridge regression best linear unbiased prediction	RR-BLUP	5-Fold CV	R package ‘sommer’
Bayes Class	Bayesian A	BayesA	5-Fold CV	R package ‘BGLR’
	Bayesian B	BayesB	5-Fold CV	R package ‘BGLR’
	Bayesian C	BayesC	5-Fold CV	R package ‘BGLR’
	Bayesian ridge regression	BRR	5-Fold CV	R package ‘BGLR’
Machine Learning	Gradient boosting decision tree	GBDT	5-Fold CV	Python3
	Random forest	RF	5-Fold CV	Python3
	Kernel ridge regression,	KRR	5-Fold CV	Python3
	Ridge regression	RR	5-Fold CV	Python3
	Elastic net regression	EN	5-Fold CV	Python3
	Multiple kernel ridge regression based on reproducing kernel Hilbert space	MKRKHS	5-Fold CV	Python3
	Light gradient boosting machine	LightGBM	5-Fold CV	Python3
Deep Learning	Deep neural network Gaussian process	DNNGP	5-Fold CV	DNNGP

### Genome collinearity and structural variation detection

To further validate the quality of genome assembly, we assessed genome collinearity using McScan [74] on the DHY, *P. trichocarpa* v4.1 (Ptr) [23], *P. deltoides* ‘I-69’ (I-69) [24], and *P. deltoides* ‘WV94’ (WV94, http://phytozome.jgi.doe.gov). Furthermore, using *P. trichocarpa* v4.1 as a reference, we conducted whole-genome comparisons to identify structural variations (SVs) using MUMmer [75], and correspondingly adjusted the orientation of chromosomes (positive or negative strands) for SVs visualization. Detected SV types encompassed translocations (≥10 kb) (TRAN), inversions (≥1 kb) (INV), duplications (DUP), insertions (INS), and deletions (DEL). Large-scale SVs were visualized using syri [76] and plotsr [77]. Bedtools [78] was utilized to identify the genes affected by SVs, and subsequent GO and KEGG enrichment analyses were performed to evaluate their functional implications.

### Whole-genome resequencing and genotyping of 765 trees

Leaf tissues from 765 trees of the reference and breeding populations were collected for DNA extraction. Genomic DNA paired-end libraries were prepared in accordance with Illumina's standard protocols and subjected to whole-genome resequencing via the Illumina HiSeq 2500 platform. Raw reads were filtered using fastp software with default parameters to generate high-quality clean data, which were subsequently aligned to the genome using BWA software. To determine the most suitable reference genome, four candidate genomes (DHY, Ptr, I-69, and WV94) were selected for mapping rate evaluation of the resequencing samples. The mapping rate results were further evaluated to determine the most optimal reference genome for subsequent analysis. Single-nucleotide polymorphism (SNP) variants were called and filtered using GATK v4.2.5.0 [79], applying standard filtering parameters. To ensure the accuracy of the markers, VCFtools v0.1.16 [80] was employed to further control the genotype data for subsequent genome-wide association studies (GWAS) analysis. The quality control standards included removing SNPs with detection rate < 0.02, quality ≤30, minimum allele frequency < 0.05, minDP <3, and those significantly deviating from Hardy–Weinberg equilibrium. Subsequently, the missing genotypes were imputed using BEAGLE [81] for subsequent genomic selection (GS) analysis.

### Population structure and linkage disequilibrium

To clarify the genetic structure of the genomic selection population for *P. deltoides*, we conducted population structure analysis based on 765 trees genotype data using the admixture and carried out principal component analysis (PCA) using GCTA software [82],. A neighbor-joining phylogenetic tree was constructed usingPHYLIP [83] with 1000 bootstrap replicates, and the results were visualized with iTOL [32]. These methods were also applied to the reference population to clarify structural differences between the breeding populations. Additionally, linkage disequilibrium (LD) decay distance was assessed using PopLDdecay v3.30 [84].

### GWAS for growth and wood property traits and QTNs identification

Based on genotype and phenotype data from the reference population, we carried out GWAS using the IIIVmrMLM software [85]. The IIIVmrMLM method encompasses two main steps: (1) genome scanning, where SNP loci with a significant *P*-value (*P* < 0.01) from the Wald test are retained for further analysis; (2) estimation of loci effects, all SNP loci retained in step 1 are incorporated into the multi-locus model, and effects were estimated using empirical Bayes. Finally, loci with a likelihood ratio test logarithm of odds (LOD) score greater than 3.0 are outputted. For significant quantitative trait nucleotides (QTNs), the threshold is set at *P* < 0.05/n, while suggestive QTNs were defined by a threshold of LOD > 3.0 [85], where *n* represents the number of SNPs [52]. Based on the population’s LD level, genes located within 16 kb upstream or downstream of the QTNs are regarded as candidate genes associated with the traits.

### Evaluation of genomic selection statistical models

To systematically conduct GS breeding for growth and wood properties of *P. deltoides*, based on the genotype and phenotype data of the reference population, we evaluated the predictive accuracy of 14 widely used GS models (Table 3). These models encompassed two BLUP models (RR-BLUP and GBLUP), four Bayesian models (BayesA, BayesB, BayesC, and BRR), seven machine learning models (GBDT, FR, SVR, RR, EN, MKRKHS, and LightGBM), and a deep learning model (DNNGP).

The assessment of model PA was performed using five-fold cross-validation, where the closer the correlation coefficient between the true phenotypic data of validation set and the GEBV values is to 1, the higher the model’s PA. To reduce the impact of random error, five-fold cross-validation was repeated 10 times, and the average PA from these repetitions was regarded as the final result. To avoid model overfitting due to excessively high marker density, we systematically evaluated PA under six random marker densities (N, 0.5 N, 0.2 N, 0.1 N, 0.05 N, 0.01 N). For each trait, the GS model that performed best across the six random marker densities was selected as the most suitable statistical model.

### Evaluation of GS model PA under different marker selection strategies

Based on the optimal model for each trait, we assessed the impact of different cross-validation methods on PA. Further, we probed into the effects of various marker selection strategies on PA and identified the optimal strategy for each trait. Three strategies were employed for marker selection: LD pruning, correlation between markers and traits, and random sampling, which yielded different marker subsets (Table 4). LD pruning was performed using Plink v2.1 [86] (parameter: --indep-pairwise 50 50 0.2), marker-trait correlations were determined based on GWAS results, and random markers were generated using vcflib [87]. Notably, to avoid model overfitting, when using GWAS to determine marker-trait correlations, GWAS should be performed only on the training set within each cross-validation fold, and not on the entire dataset.

**Table 4:** Genomic selection marker selection strategies

Selection Strategy	Objective	Approach	CV	Repetitions
LD pruning	Assess the impact of marker LD levels on PA	LD ≤ 1, LD ≤ 0.8, LD ≤ 0.5, LD ≤ 0.3, LD ≤ 0.2, LD ≤ 0.1	5-Fold	10
Marker-trait correlation	Assess the impact of marker-trait correlation on PA	*P* ≤ 1, *P* ≤ 0.5, *P* ≤ 0.1, *P* ≤ 0.01, *P* ≤ 0.001, *P* ≤ 0.0001	5-Fold	10
Random	Serve as a control for LD and GWAS *P*-value assessments	Generate different random markers as controls	5-Fold	10

### Breeding populations genomic prediction and seedling growth traits validation

Based on the optimal statistical model and marker subset, genomic prediction (GP) for the breeding population was conducted using GWAS-significant associations as covariates. The GEBVs for each trait were utilized to rank the population, and the top 20% were selected as excellent clones. Subsequently, a kinship network was established based on the genetic background of the outstanding clones to identify core parents for the genetic improvement of *P. deltoides*. To assess the accuracy of GP for growth traits, the Pearson correlation coefficient between seedling growth observations and GEBVs was computed.

### Transcriptome sequencing of extreme individuals for GP

We calculated the genomic composite breeding values (GCBVs) for growth traits, with an equal weight of 0.5 assigned to both H and DBH. Subsequently, we combined GCBVs with seedling growth data to select extreme individuals to collect developing xylem tissue for transcriptome sequencing. Differentially expressed genes were identified using edgeR [88]. The aim was to validate the accuracy of GP and identify the final candidate genes by integrating GWAS association results.

## Supplementary Material

Web_Material_uhaf165

## Data Availability

DHY genome sequencing data have been deposited under National Genomics Data Center (https://ngdc.cncb.ac.cn/?lang=en) under BioProject PRJCA033198. The whole-genome resequencing data have been deposited under BioProject PRJCA033212. The transcriptome sequencing data have been deposited under BioProject PRJCA033213. All other data related to this study are provided in the supplementary files of the article.
