# Supplementary material for: Genomic selection for growth and wood properties in multi-generation hybrid populations of Populus deltoides
Source: Hortic Res. 2025 Jun 25;12(9):uhaf165. doi: 10.1093/hr/uhaf165 (PMC12342176; doi:10.1093/hr/uhaf165)
Supplement: Web_Material_uhaf165 [file web_material_uhaf165.zip › Supplemental FigureS1-S15.docx]

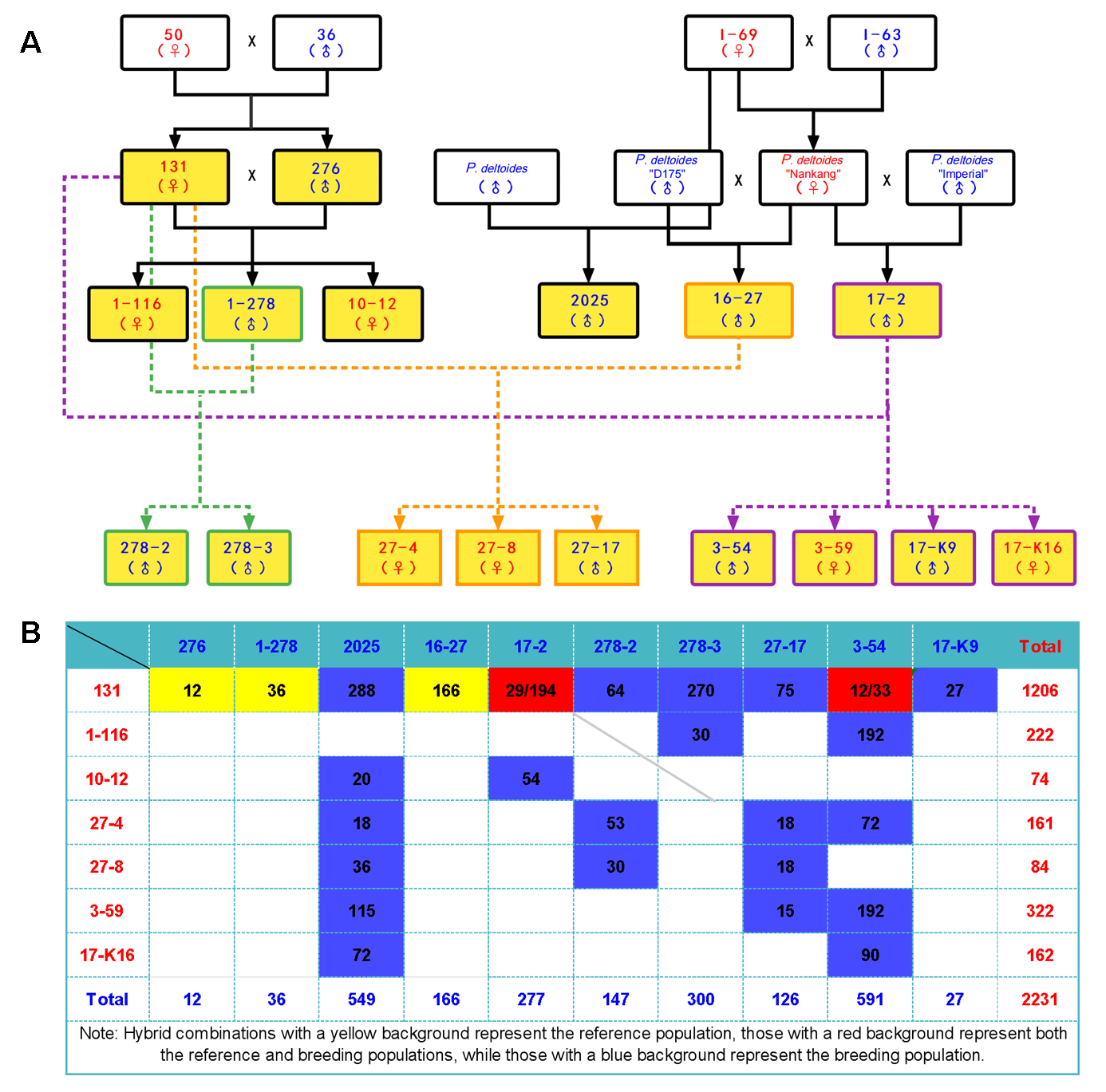


**Supplementary Figure S1. Genetic relationships among breeding parents and the number of hybrid offspring per cross.** (A) Genetic relationships among breeding parents; 131: *P. deltoides* 'Danhong' (DHY); 2025: *P. deltoides* '2025'; 3-59: *P. deltoides* 'Zhongcheng 3'; 3-54: *P. deltoides* 'Zhongcheng 4'; 1-116: *P. deltoides* 'Zhongcheng 5'; 16-27: *P. deltoides* 'Beiyang'; 17-2: *P. deltoides* 'Chuangxin'; 276: *P. deltoides* 'Nanyang'; 1-278: *P. deltoides* 'Zhongcheng 2'. (B) The number of hybrid offspring per cross.


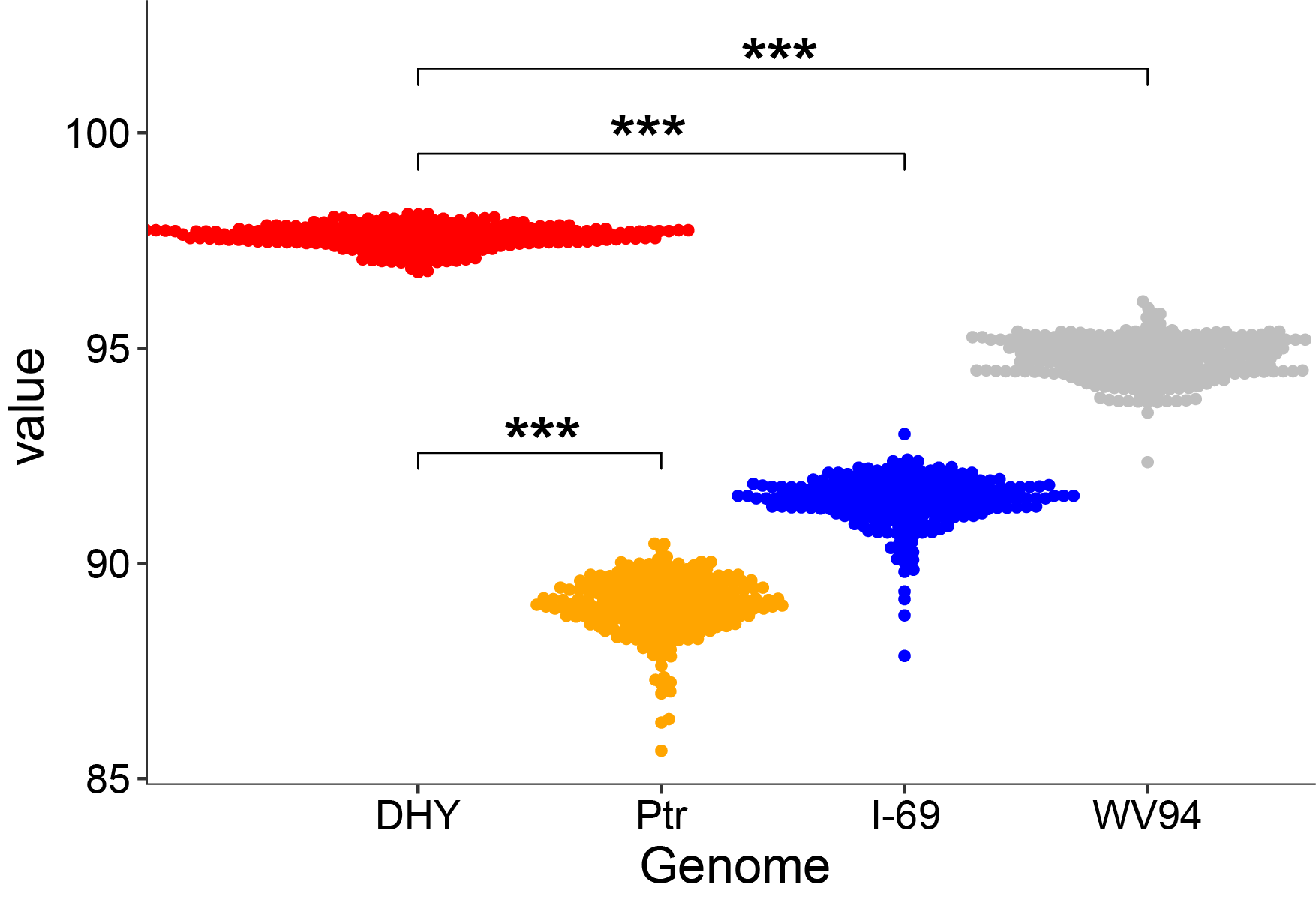


**Supplementary Figure S2. Mapping rate of whole-genome resequencing data for the reference population.**


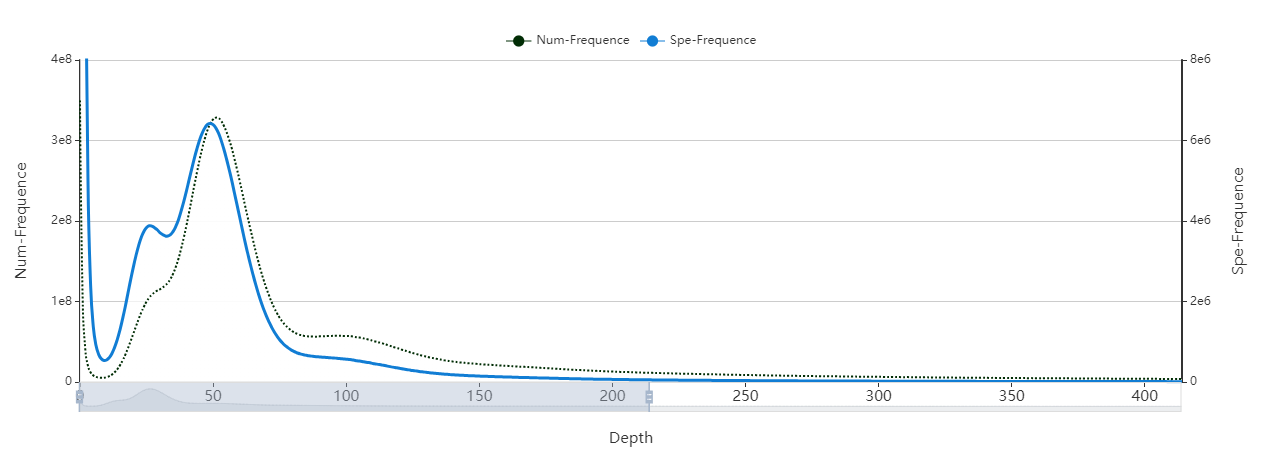


**Supplementary Figure S3. Depth and K-mer count and frequency distribution.**


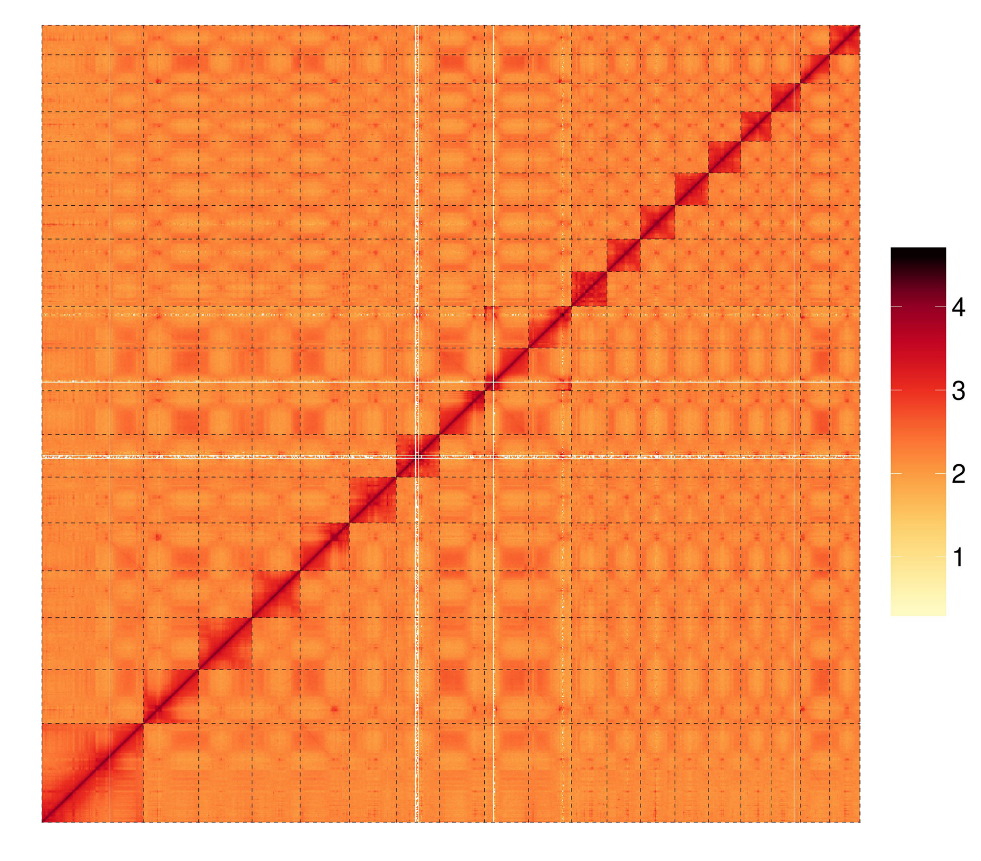


**Supplementary Figure S4. Hi-C heatmap of chromosomal interactions, with the x-axis and y-axis arranged by chromosome length.** The blank regions observed in the Hi-C heatmap are primarily located near the centromeres and are composed of long repetitive sequences. There are no gaps in the genome sequence, and the corresponding chromosomes are gap-free.


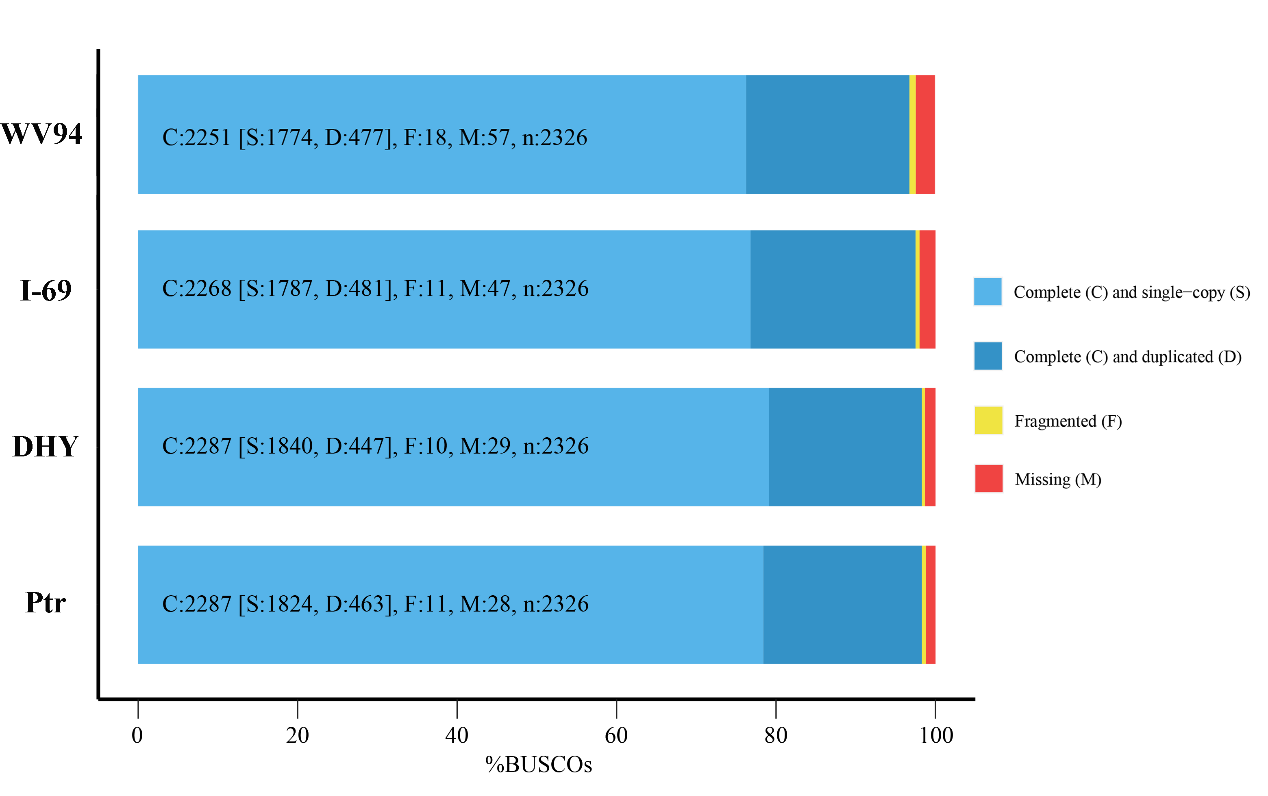


**Supplementary Figure S5. BUSCO assessment results of the four genomes, evaluated using the 2 326 orthologous genes from the eudicotyledon database.**


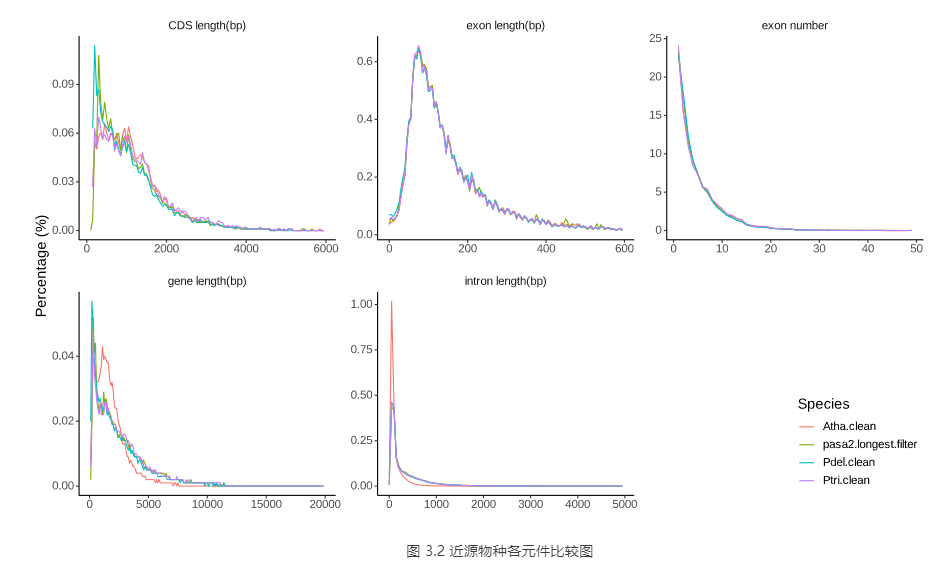


**Supplementary Figure S6. Comparative diagram of various elements among closely related species.**


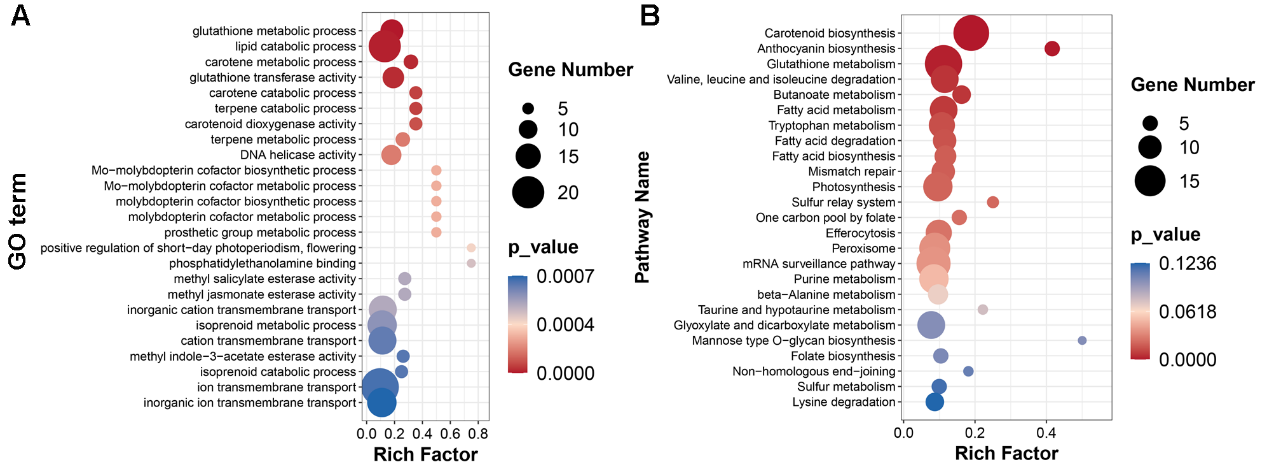


**Supplementary Figure S7.** **GO and KEGG enrichment analysis of genes affected by shared structural variations in *P. deltoides*.**

(A) GO enrichment analysis bubble plot. (B) KEGG enrichment analysis bubble plot.


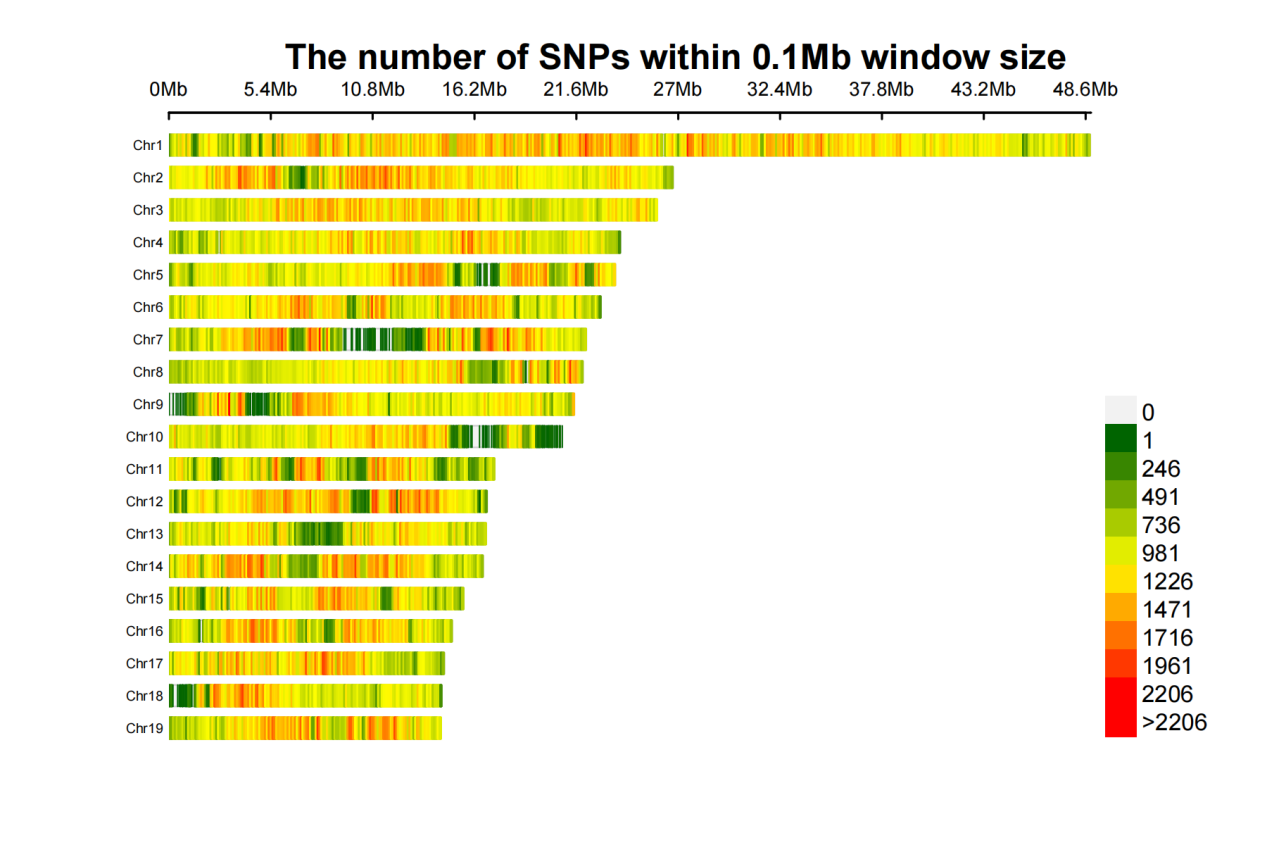


**Supplementary Figure S8. Distribution of high-quality SNP markers across the DHY genome.**


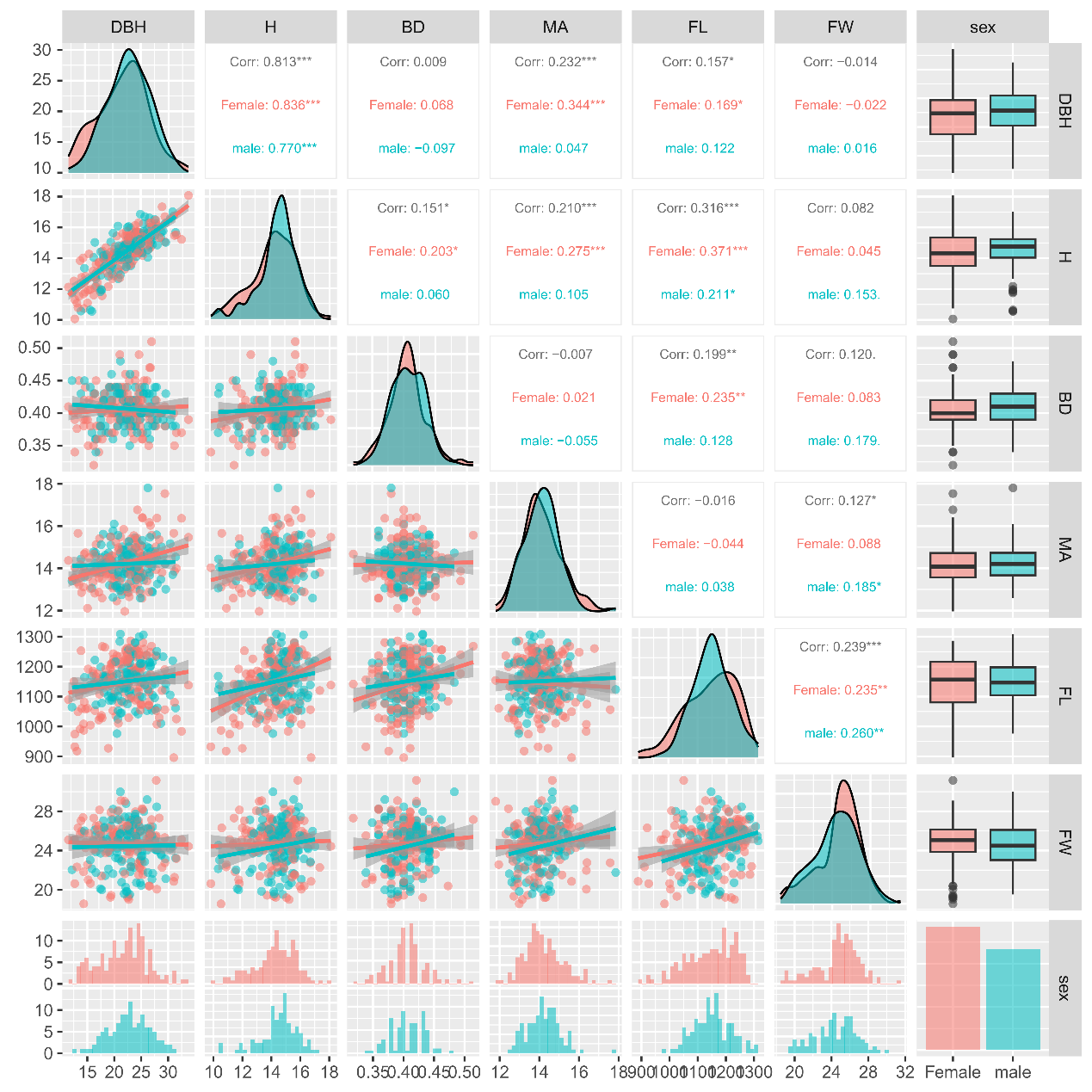


**Supplementary Figure S9. Distribution and correlation of growth and wood property traits.**


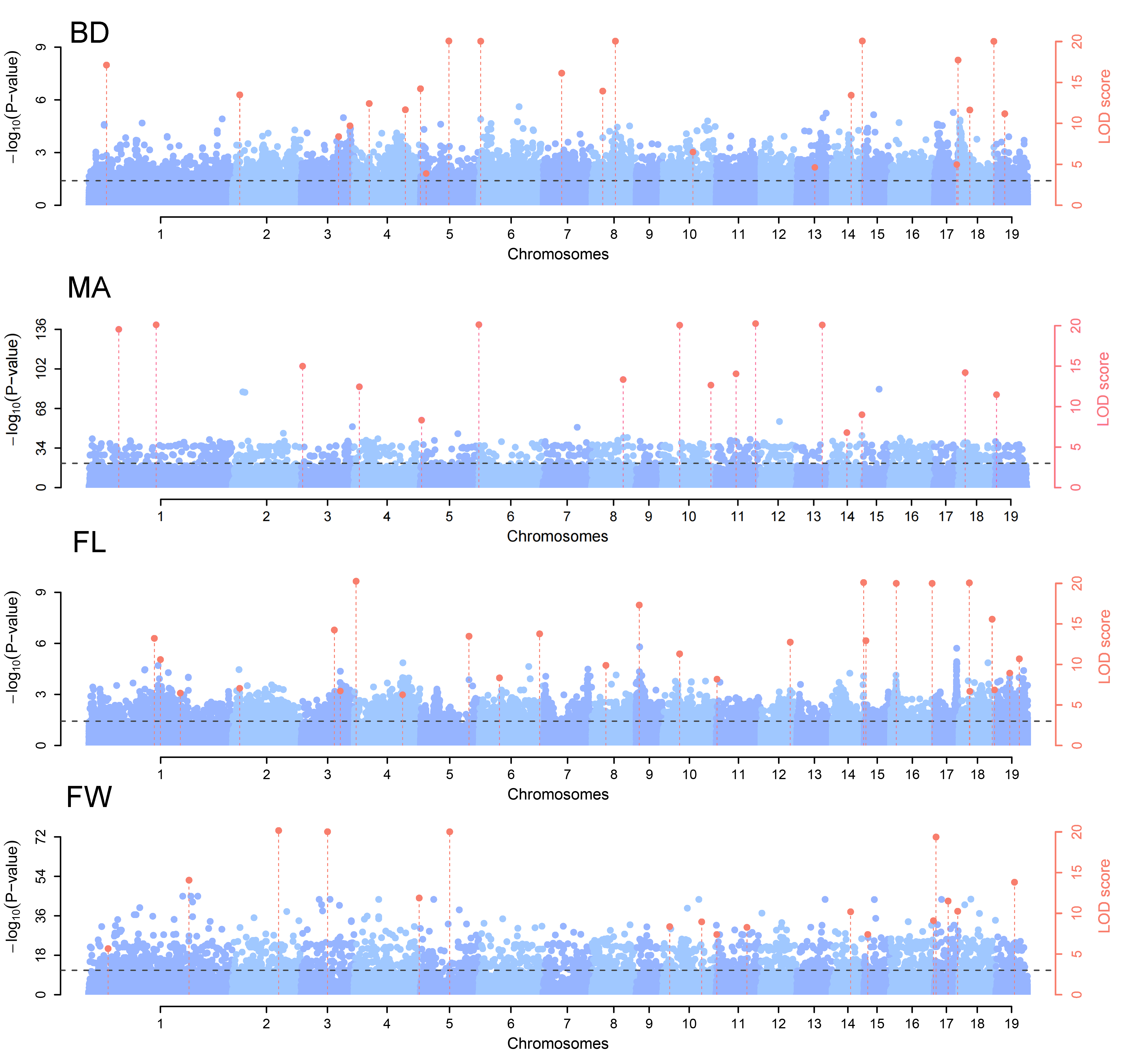


**Supplementary Figure S10. Manhattan plots of GWAS associations for wood property traits.** The left Y-axis shows the -log10 P values of all SNP obtained from the first step of single-marker scanning; the right Y-axis shows the LOD values of all significant and suggestive loci from the second step. The dashed line indicates the threshold for the second round of detection using the IIIVmrMLM model (LOD = 3).


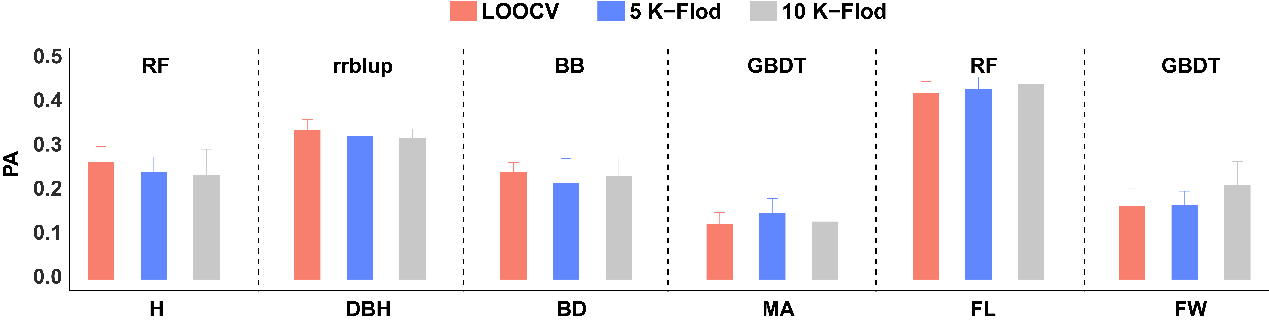


**Supplementary Figure S11. PA evaluation results under the optimal model using different cross-validation methods.**


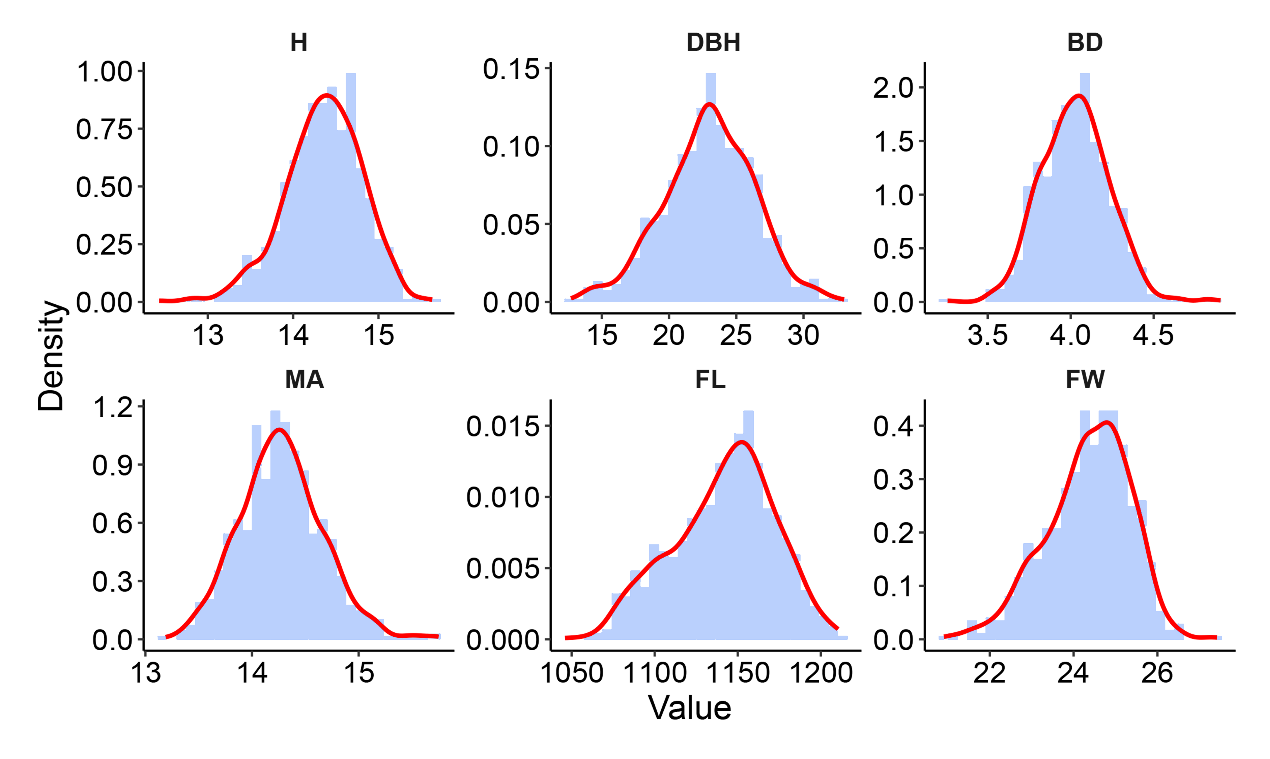


**Supplementary Figure S12. Normal distribution curves of GEBV for six traits.**


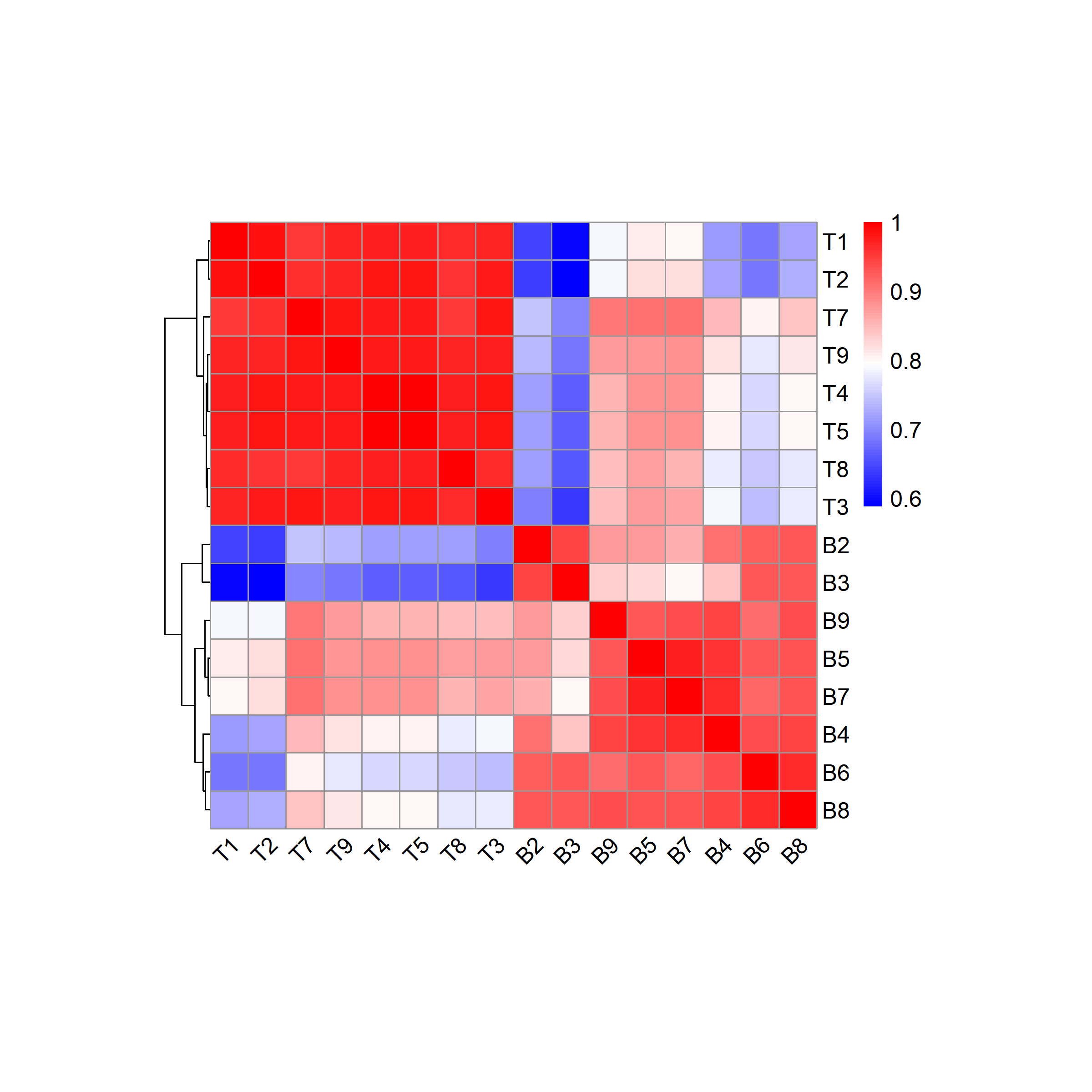


**Supplementary Figure S13. Clustering heatmap of transcriptome samples.**


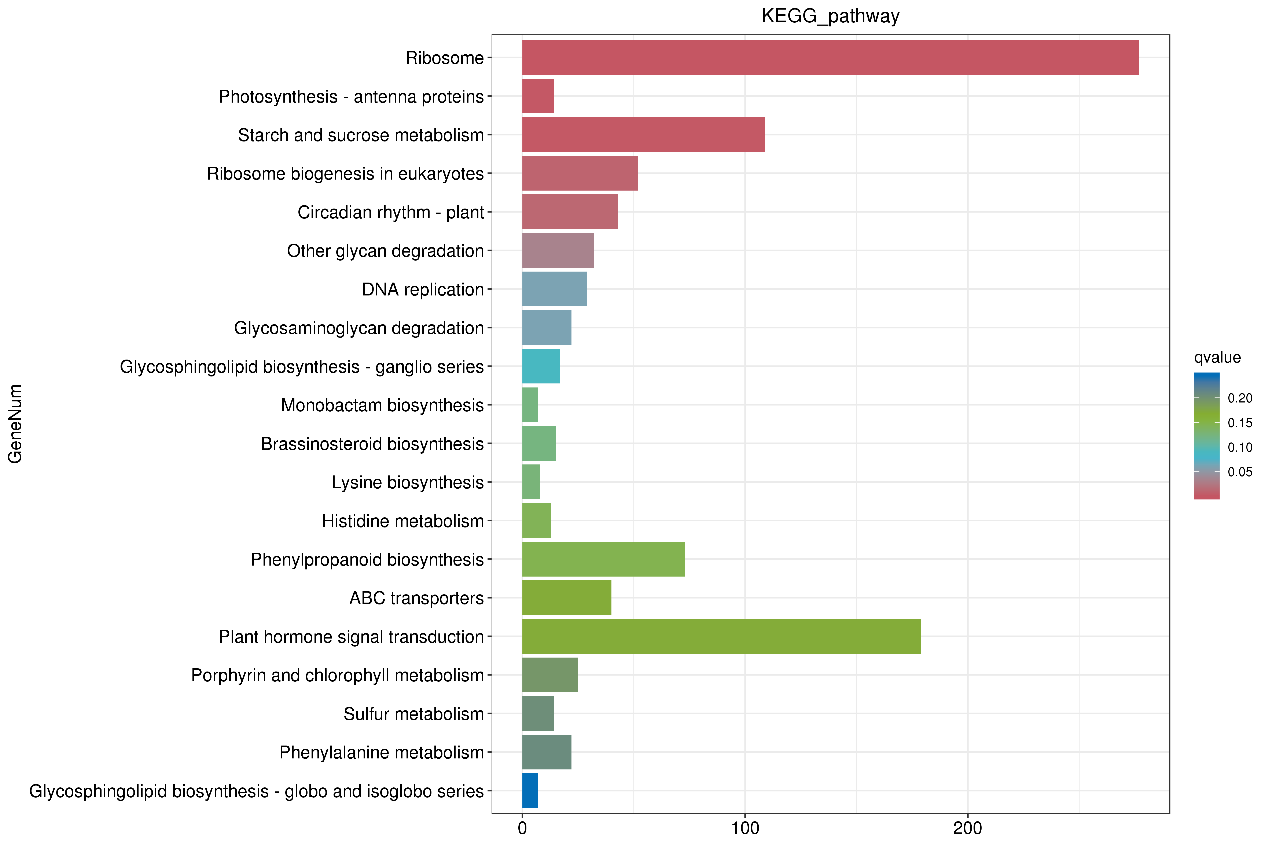


**Supplementary Figure S14. KEGG enrichment analysis results of differentially expressed genes.**


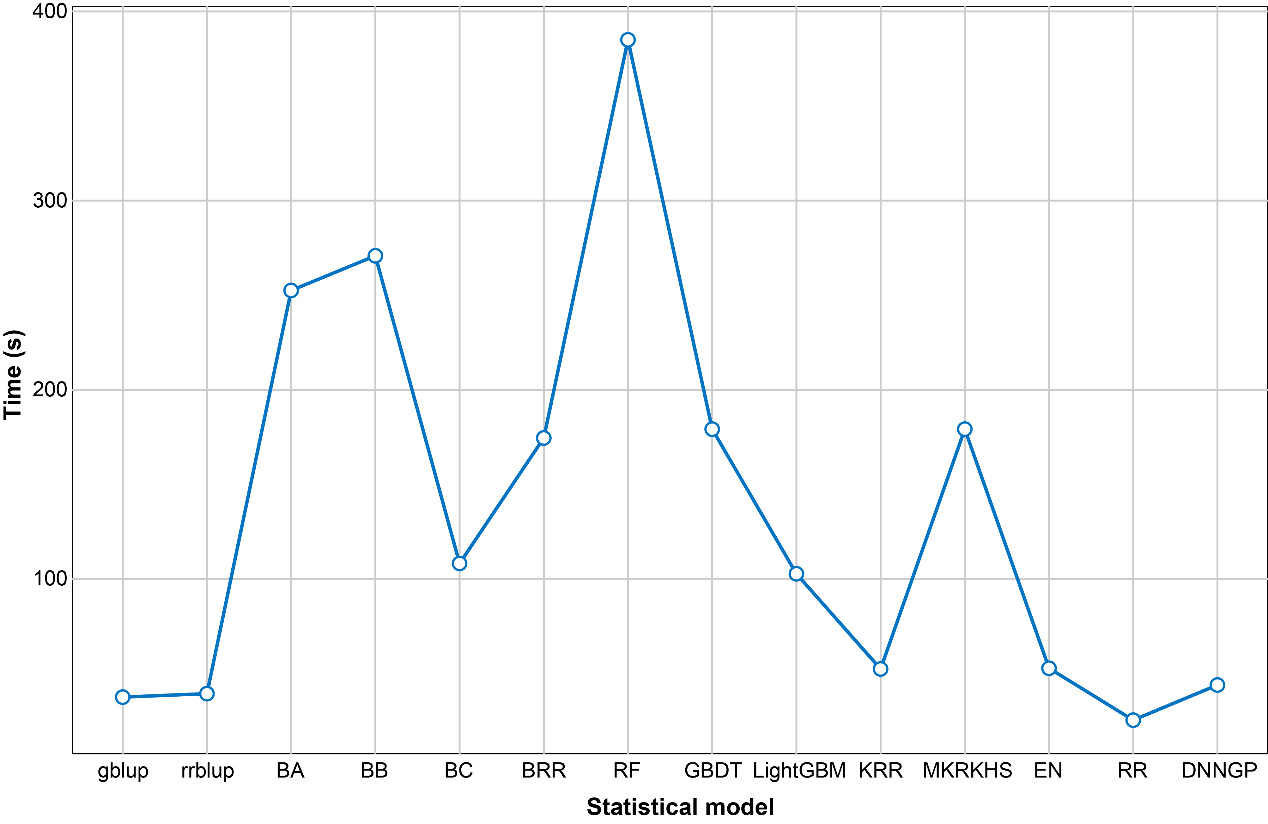


**Supplementary Figure S15. Time required by different GS statistical models, as illustrated by a line chart.** The markers used were filtered with LD=0.2.
